# Genetic Potential for N₂O Metabolism in Tree Tissues: Insights From Nitrogen Cycling Gene Prevalence and *nosZ* Diversity Across Tree Species

**DOI:** 10.1007/s00248-026-02773-8

**Published:** 2026-04-18

**Authors:** Krishnapriya Thiyagarasaiyar, Dhiraj Paul, Johanna Kerttula, Milja Keski-Karhu, Kaido Soosaar, Ülo Mander, Sara Hallin, Katerina Machacova, Jukka Pumpanen, Henri M.P. Siljanen

**Affiliations:** 1 Department of Environmental and Biological Sciences, University of Eastern Finland, Kuopio, Finland; 2 Department of Geography, Tartu University, Tartu, Estonia; 3 Department of Forest Mycology and Plant Pathology, Swedish University of Agricultural Sciences, Uppsala, Sweden; 4 Science for Life Laboratory, Swedish University of Agricultural Sciences, Uppsala, Sweden; 5 Department of Ecosystem Trace Gas Exchange, Global Change Research Institute of the Czech Academy of Sciences, Brno, Czech Republic

**Keywords:** Tree-microbiome, *NosZ* diversity, Nitrous oxide, Targeted metagenomic

## Abstract

Nitrous oxide (N_2_O) is a potent greenhouse gas, and microorganisms play a crucial role in its metabolism. While N_2_O cycling among soil microorganisms is well studied, there is a major knowledge gap regarding the distribution and diversity of these microorganisms within tree ecosystems. In this study, we aimed to comprehensively assess the potential for nitrogen (N) cycling and the diversity of N_2_O-reducing microorganisms in shoots (leaves and terminal branches) and wood cores of four tree species — European beech (*Fagus sylvatica*), European hornbeam (*Carpinus betulus*), birch (*Betula pendula* and *Betula pubescens*) and Norway spruce (*Picea abies*). We assessed N_2_O exchange through shoot incubation experiments and measured internal N_2_O concentrations in stem wood. Inorganic N species were studied as indicators of microbial transformation, and a targeted metagenomic approach was used to determine the relative abundance of N-cycling genes and *nosZ* clade I and II diversity. Our study revealed that hornbeam shoots showed potential N_2_O emissions (0.002–0.007 ng N_2_O g⁻¹ FW h⁻¹), while beech shoots indicated N_2_O consumption (-0.001 to -0.017 ng N_2_O g⁻¹ FW h⁻¹). Birch had internal stem wood N_2_O concentration of + 150.39 ppb, and beech − 9.74 ppb when compared to the ambient concentration. Targeted metagenomic analysis revealed the presence of key nitrification and denitrification genes in both tissue types. In particular, *nosZ* genes were detected in shoots (0 to 26.48 per 100,000 reads) and in wood cores (0 to 31.95 per 100,000 reads), with clade I dominating over clade II and *Rhizobiales* prevalent within clade I. Overall, our findings show that internal tree tissues harbour distinct N‑cycling microbial assemblages dominated by *nosZ* clade I, suggesting that trees may function as localized N_2_O sinks or sources depending on tissue type and microbial composition.

**Supplementary Information:**

The online version contains supplementary material available at 10.1007/s00248-026-02773-8.

## Introduction

Nitrous oxide (N_2_O) is a potent greenhouse gas with a global warming potential about 300 times greater than CO_2_ over a 100-year horizon. Since the preindustrial era, atmospheric N_2_O concentrations have increased from 275 to 338 parts per billion (ppb), largely driven by anthropogenic activities such as the application of synthetic nitrogen (N) fertilizers, cultivation of N_2_-fixing crops, and fossil fuel combustion [1, 2]. Land-use changes, including wetland drainage, also contribute to elevated N_2_O emissions [3, 4]. Microbial production of N_2_O through denitrification and nitrification in soils is considered the dominant source of N_2_O entering the atmosphere [5]. However, significant uncertainty remains in global N_2_O budget estimations due to the limited understanding of other potential sources and sinks [6]. Recent studies suggest that plants can also act as significant sources and sinks of N_2_O [7, 8]. Yet, the microbial pathways and organisms responsible for N_2_O production and consumption within tree tissues remain poorly understood, despite their critical role in shaping tree-mediated N_2_O fluxes. Addressing this gap is crucial, as it directly influences our understanding of tree-associated microorganisms and their contribution to N_2_O dynamics.

Gas flux measurements, particularly from tree stems, have been widely studied to quantify tree-mediated N_2_O exchange [7, 9–12], whereas research on phyllospheric N_2_O fluxes is only emerging [8]. Recent evidence suggests that microbial communities in the spruce phyllosphere have the potential for N_2_O exchange [13]. Further, canopy nitrification contributes significantly to forest N-cycling [14], indicating an active microbial role in these processes. Yet, the identities and functions of microorganisms involved in N_2_O metabolism across different tree species and tissues remain largely unknown. Tree tissues contain microenvironments where both aerobic and anaerobic microorganisms can occur [15], implying that microorganisms inhabiting these tissues may be involved in aerobic and anaerobic N-transformation process and mediate N_2_O production and reduction. Further insight is necessary for improving N_2_O budget estimates, as the balance between microbial N_2_O‑producing and N_2_O‑reducing pathways within trees, together with N_2_O production by tree physiological processes and the transport pathways that move N_2_O within and out of the tree, ultimately determines whether trees and forests act as net N_2_O sources or sinks. This study addresses the gap by providing a comprehensive assessment of tree-associated microbial contributions to N_2_O dynamics across temperate, hemi-boreal and boreal forests.

The present study aims to detect and compare the microbial communities involved in N-cycling metabolism within tree shoots, which include leaves and terminal branches, as well as wood cores of four tree species. To achieve this, we utilized targeted metagenomics using probe capture [13, 16] specifically designed to identify N-cycling genes in host-microbe systems. This approach enabled us to evaluate the relative abundance of N-cycling genes, including bacterial and archaeal ammonia monooxygenase (*amoA*) and nitrite oxidoreductase (*nxrB)*, which are key genes involved in nitrification, as well as nitrite reductase (*nirK* and *nirS*), nitric oxide reductase (*norB*) and nitrous oxide reductase (*nosZ*), which are essential for denitrification. We also assessed the diversity of *nosZ* clades I and II, which play a critical role in N_2_O reduction [17], and evaluated whether tree species influence the relative abundance of N-cycling genes and distribution of *nosZ* microbial communities. To complement the DNA-based microbial data, we conducted a short-term incubation experiment to measure N_2_O exchange from shoots and analyzed internal N_2_O concentrations in stem wood, providing functional evidence of N_2_O cycling activity. We also quantified inorganic N species in tree tissues as indicators of microbial transformations. Ultimately, our study aims to enhance our understanding of the contributions of tree-associated microorganisms to N_2_O dynamics and to highlight the importance of aboveground tree-microbe interactions in forest N_2_O gas exchange. We hypothesize that shoot and wood‑core tissues may create distinct microhabitats that shape microbial nitrogen‑cycling potential, with additional variation among species that may reflect both biological and location factors.

## Methodology

### Sample Collection for N_2_O Metabolism Analysis and Nucleic Acid Extraction

Ten forest sites were included in the study, several of which have previously reported N_2_O fluxes [4, 18, 19]. Samples of shoots (leaves/needles with terminal branches) and wood cores were collected from trees representing different dominant species: European hornbeam (*Carpinus betulus* L.), European beech (*Fagus sylvatica* L.), birch (*Betula pubescens* Ehrh. and *Betula pendula* Roth.), and Norway spruce (*Picea abies* (L.) H. Karst.). For analysis, downy birch and silver birch were combined and treated as a single category (‘birch’) due to their similar ecological characteristics [20]. Sampling was conducted between April and September 2022. To improve geographical and climatic representation, tree species from different climate zones were included. Samples were collected from sites in temperate forests (Lanžhot and Štítná nad Vláří, Czech Republic), hemiboreal forests (Agali II and Kiidjärve, Estonia; Bromarv, Finland) and boreal forests (Puijo and Kenttärova-Pallas, Finland) (Table 1).

**Table 1:** Information on sampling sites, tree and soil characteristics

Species study sites	Coordinates	Site description	Forest type, Stand age	Stand height, Stem diameter at Breast height	Soil type	Soil pH	Annual precipitation (mm)	Mean Annual temperature (°C)	Stem *N*_2_O flux	Reference
Hornbeam, Lanžhot, Czech Republic (L1)	(48,68133°P, 16,94602°I)	Temperate floodplain forest	110-year-old 60% of hornbeam, 20% ash, 15% of oak, 5% of elm, maple and tilia	23.4 m, 35 cm	Eutric Humic Fluvisol, Haplic Fluvisol and Eutric Fluvisol	5.7	497	9.7	*0.24 ± 0.34 µg N_2_O m^− 2^ h^− 1^	[18, 21]
Beech, Štítná nad Vláří, Czech Republic (L2)	(49.02°N, 17.58°E)	Temperate montane upland forest	115-year-old monoculture	32.2 m, 60 cm	Eutric (Stagnic) Cambisol	7.0	800	7.5	−3.8 µg N_2_O m^− 2^ h^− 1^	[22, 23]
Beech, Bromarv Rilax manor, Finland (L3)	(59,95826°P, 23,06783°I)	Hemiboreal upland	95-year-old mono-culture	30 m, 48 cm	Inceptisols	4.2	758	NA	NA	[24]
Birch, Agali-II, Tartu, Estonia (L4)	(58°17’00.0"N 27°17’00.0"E)	Hemiboreal wetland	32-year-old, 65% of Downy birch and 35% of Norway spruce	15 m, 14 cm	Oxalis	5.9	650	4.8	*−23.69 ± 236.87 µg N_2_O m^− 2^ d^− 1^	[4, 25]
Spruce, Agali-II, Tartu, Estonia (L5)	(58°17’00.0"N 27°17’00.0"E)	Hemiboreal wetland	32-year-old, 65% of Downy birch and 35% of Norway spruce	17 m, 18.4 cm	Oxalis	5.8	650	4.8	*6.96 ± 4.51 µg N_2_O m^− 2^ d^− 1^	[4, 25]
Birch, Kiidjärve, Tartu, Estonia (L6)	(58,17896°P, 27,08408°I)	Hemiboreal upland	31-year-old, monoculture, Birch	19–24 m, 16 cm	Alfisol, Sandy	5.2	NA	NA	NA	[26]
Birch, Puijo, Kuopio, Finland (L7)	(62.908328, 27.659551)	Boreal upland	165-year-old 90% spruce, 10% birch	18–22 m, 22 cm	Podlozied soil	3.4	549	5	NA	
Spruce, Puijo, Kuopio, Finland (L8)	62°54′N, 27°39′E)	Boreal upland	165-year-old 90% spruce, 10% birch	24–29.5 m, 41–68 cm	Podlozied soil	3.2	549	5	NA	[27]
Spruce, Kenttärova, Pallas, Finland (L9)	(N67°59.237’, E24°14.579’)	Subarctic upland	70–160-year-old mono culture spruce	13 m, 18 cm	Podzol	4.4	484	−1.4	NA	[28]

*Data from this study; tree stem N_2_O fluxes were measured at selected sites (Lanzhot, Czech Republic and Agali‑II, Estonia), following the approaches described in [7] and [22], respectively

**Fig. 1:**
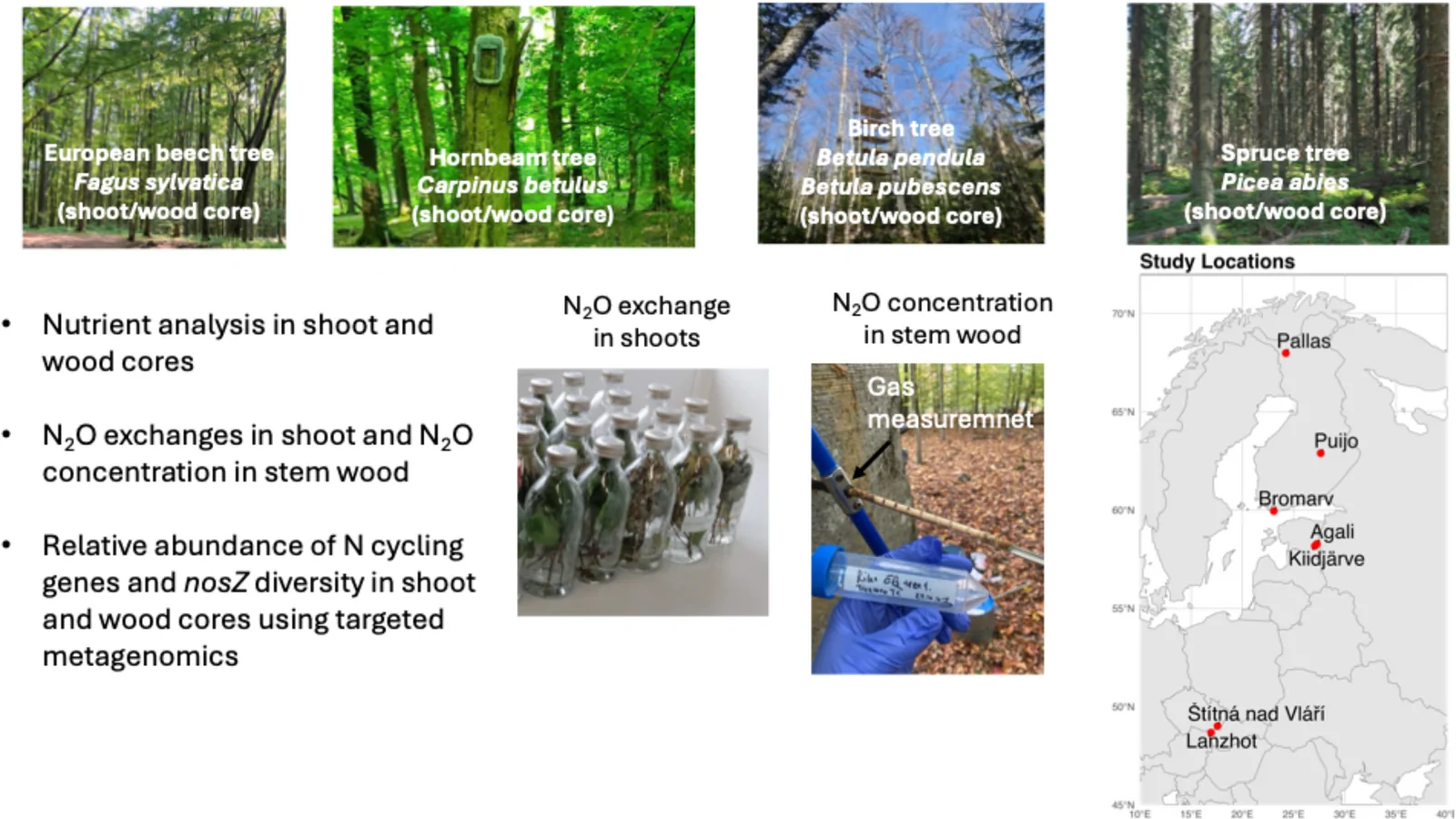
Description of sites and techniques used in this study

We collected one shoot and one wood core sample from each of three replicate trees (*n* = 3) per species at each site. All samples were collected from the north side of the tree. The standard sampling height was approximately 1.5 m, except for the birch shoot samples from Agali, where the lowest branches were at around 10 m height, and the beech and hornbeam shoot samples from the Czech Republic site, which were collected at approximately 6 m height using scissors attached to a long, telescopic handle. Wood cores were taken using a 5 mm increment borer (Haglöf, Sweden) at a height of 1.5 m. The length of the wood cores was 5–7 cm for both birch and spruce woods and 8–10 cm for beech woods. Differences in core length were related to differences in stem diameter. Samples of wood cores and shoots that were used for DNA extraction were immediately taken into 50 mL sterile Falcon tubes and instantly snap frozen with liquid N_2_, and stored at − 80 °C. In addition, extra samples of shoots and wood cores were taken and separated into plastic bags (2 L) for further analysis (Fig. 1).

## N_2_O Exchange Measurements for Shoots

A small incubation experiment was conducted using detached shoots to evaluate their potential for N_2_O exchange and provide a functional context for metagenomic data. For all sampling locations, shoot incubations were initiated within 20 min after detachment. Approximately 10–20 g of fresh weight (FW) shoots were placed in 500 mL glass bottles containing 50 mL of 0.9% NaCl solution to maintain osmotic pressure, ensuring the solution touched the branches. Bottles were sealed with rubber septa and aluminum crimp caps, then supplemented with 120 mL of ambient indoor air. Incubation was carried out under controlled conditions: 12 h in light (photosynthetically active radiation, PAR 300 µmol m⁻² s⁻¹) at 15 °C, followed by 12 h in darkness at 4 °C. Blank bottles without plant material served as controls under identical conditions. Gas samples (20 mL) from the incubation bottles (samples and blank) were taken at 1, 24 and 72 h from the start of the incubation using polypropylene syringes (BD Plastipak™; Becton, Dickinson, and Company equipped with three-way stopcocks valve connected syringe needles (0.8 × 40 mm) (BD Precisionglide^®^) and transferred to pre-evacuated 12 mL Labco vials flushed with N_2_. The CO_2_ and N_2_O concentrations from shoots were measured using an Agilent 7890B Gas Chromatograph (GC) (Agilent Technologies, Palo Alto, CA, USA) equipped with Gilson liquid handler GX271 autosampler (Gilson Inc., Middleton, WI, USA) and a Hayesep Q 80/100 mesh column and an electron capture detector (ECD) [29]. The method detection limit (MDL) was calculated according to USEPA guidelines. Seven blank replicates produced a standard deviation of 3.80 × 10⁻⁴ ng N_2_O g⁻¹ h⁻¹, resulting in an MDL of 1.19 × 10⁻³ ng N_2_O g⁻¹ h⁻¹. The N_2_O production or consumption potential rates over the incubation period were calculated using the ideal gas formula (Eq. 1).

1 $$N_2O\;exchanges=\left(\frac{V\triangle CPM}{RTW}\right)\left(\frac1{\triangle t}\right)$$

 

Where V= volume of gas phase in the incubation bottle (mm^3^), ΔC = change in concentration of gas (ppm), P = air pressure (Pa), M = molecular mass of the gas (g mol^− 1^), T = temperature (K), R = universal gas constant (8.314 J mol^− 1^ K^− 1^), W=weight of sample (g), Δt = change in time (h).

Although the controlled incubations of shoots cannot replicate real-time fluxes, they provide a standardized and comparable way to assess the potential for N_2_O production and consumption across species and sites. Sterilized controls were not included because sterilization alters tissue structure, moisture content, and gas movement, making treated samples unsuitable for comparison with natural tissues. Therefore, our incubation results should be interpreted as N_2_O exchange potentials rather than precise estimates of biological N_2_O production or reduction.

## N_2_O Concentration Measurements in Stem Wood Bore Holes

N_2_O concentration in stem wood bore holes was measured using the previously described method [30]. After collecting wood cores, the increment borer was resealed to create an airtight chamber for gas measurements. Ambient concentration served as a reference for comparing stabilized N_2_O levels in wood bore holes. After 5 min, 20 mL gas samples were withdrawn from the sealed borer using syringes and injected into pre-evacuated, N_2_-flushed glass vials (Labco Limited, Lampeter, UK). N_2_O concentration in the stem wood was measured using gas chromatography equipped with ECD [29]. The detection limit for the GC analysis was calculated as 3 times the SD of the N_2_O standard gas concentration, which is 12 ppb. Because ambient N_2_O concentrations vary across sites, N_2_O concentrations in stem wood were compared with ambient air to determine the difference in N_2_O concentrations in stem wood. Negative values indicate lower N_2_O inside the wood than outside (potential consumption), while positive values indicate higher internal concentrations (potential production).

## Analysis of Nutrient Content in Tree Tissues

Shoots and wood cores were snap-frozen in liquid N_2_, ground with a sterile mortar and pestle (Haldenwanger, Berlin, Germany) using liquid N_2_, and stored in 50 mL Falcon tubes at − 80 °C. Tree tissues (1 g) were extracted in 1 M KCl (3 mL), shaken for 1 h at 175 rpm (Heidolph, Schwabach, Germany), then centrifuged at 13,000 rpm for 5 min (Eppendorf, Horsholm, Denmark) for nutrient analysis, such as ammonium (NH_4_⁺), nitrite (NO_2_⁻), and nitrate (NO_3_⁻). Supernatants were filtered through PES membrane filters (0.22 μm; Merck KGaA, Darmstadt, Germany) and stored at − 20 °C until spectrophotometric analysis as described previously.

## DNA Extraction

Community DNA was extracted from ground shoots and wood cores using DNeasy^®^ PowerSoil^®^ Kit (Qiagen, Hilden, Germany) with minor modifications [31]. Briefly, 0.1–0.2 g of sample was homogenized in CD1 solution using Fastprep (Savant Fast Prep FP120 Bio 101, USA) for 30 s (2×) at 5.5 m s⁻¹ and vortexed for 10 min. After adding CD3 solution, samples were incubated for 1 h before continuing the protocol. DNA quality and concentration were checked using NanoDrop Lite (NanoDrop Technologies, Wilmington, NC, USA) and Qubit 4 fluorometer (Thermo Fisher Scientific, Waltham, MA, USA). After DNA extractions, both shoot and wood core samples (*n* ≥ 3**)** from each tree species were sent for targeted metagenomics (Arbor Biosciences, Arbor, Michigan, USA).

### Targeted Metagenomics with Probe Capture and Bioinformatic Analyses

To investigate the presence and diversity of tree-associated microorganisms capable of N_2_O reduction, a probe capture approach followed by shotgun sequencing was used. This method, more sensitive than traditional metagenomics [16], enabled the detection of low-abundance microbial genes within the host-microbe ecosystem [13, 32] as plant DNA remains largely unsequenced.

A curated target gene database of nitrification and denitrification genes for probe production was compiled using GenBank, BLAST, and HMM-based searches [16]. Gene-specific probes were designed using the MetCap pipeline [33, 34], resulting in 263,111 unique probes. These were synthesized with biotin labelling (myBaits Custom-kit, Daicel Arbor Biosciences, Michigan, USA) for streptavidin-coated magnetic bead-based purification. DNA samples were fragmented, adapter-ligated, and hybridized with probes at 47 °C for 72 h. Post-hybridization, libraries were purified and pooled for sequencing on the Illumina NovaSeq platform with PE150 chemistry. The DNA libraries, probe hybridization and sequencing were done in myReads service of Daicel Arbor Biosciences (Arbor, Michigan, USA). Raw reads were quality-checked with FastQC and trimmed using Trimmomatic (Q > 30). To accurately identify functional genes, we employed advanced HMMER profiles [16, 35] to meticulously search for sequences corresponding to each target gene against the gene-specific database, setting maximum E-value cut-off (E < 0.0001). Gene abundances were normalized to the total read counts of all captured genes. Thus, reported abundances represent relative representation of target genes within the captured gene pool. Following this, we used graftM in conjunction with the Gappa tool, based on a robust *nosZ* reference phylogeny [36], to determine the taxonomic affiliations and relative abundances of *nosZ* clade I and clade II as previously described [37].

### Statistical Analysis

Data normality was assessed using the Shapiro-Wilk test. Normally distributed data were analyzed with one-way ANOVA and Tukey HSD, while non-normal data used Kruskal-Wallis with Dunn’s test and Bonferroni correction. These analyses were performed on datasets presented based on location, as shown in the supplementary (Figs. S1–S7). Pairwise comparisons between shoot samples and blank controls were performed using the Wilcoxon rank‑sum test. Generalized linear mixed models were applied to N‑cycling gene abundances and N contents because their residuals did not meet normality assumptions, while linear mixed models were used for N_2_O exchange measurements and $\:\varDelta\:\:$N_2_O concentrations in stem wood. To address potential confounding by location, all models were fitted with tree species as the fixed effect and location as a random effect, and we report effect sizes, 95% confidence intervals, and false discovery rate (FDR)-adjusted *P*‑values using libraries lme4, performance, emmeans [38–40]. Significant differences between tree species (hornbeam, beech, birch, spruce) and tree tissues (shoot, wood cores) were tested with robust ANOVA via the Aligned Rank Transform method [41]. Spearman correlations were computed and visualized with corrplot. All analyses were performed in R (v4.4.0).

## Results

### Nutrient Content in Shoots and Wood Cores

**Fig. 2:**
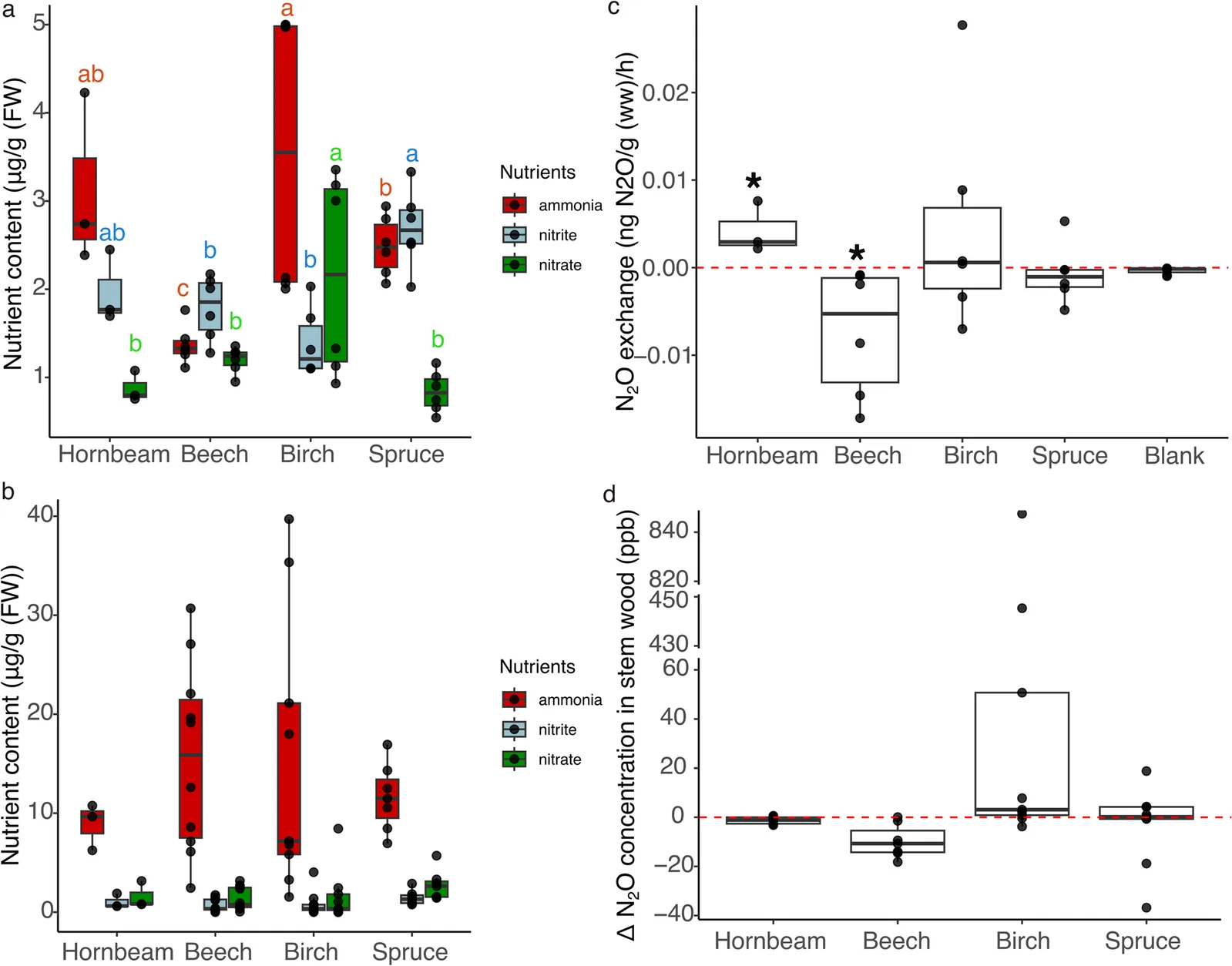
Nutrient content and N_2_O dynamics in tree tissues collected from different tree species. a: NH_4_^+^, NO_2_^−^ and NO_3_^−^ contents in shoots, b: NH_4_^+^, NO_2_^−^ and NO_3_^−^ in wood cores, c: N_2_O exchange of shoots, d: Δ N_2_O concentration in stem wood bore holes (C_stem wood_ – C_ambient air_). Boxplots showed individual data points (black dots) and median values (black line; *n* ≥ 3). Significant differences are indicated by different letters for each nutrient across tree species in panel a (FDR-adjusted *P* < 0.05; pairwise comparison of estimated marginal means, Table S1). In panel c, asterisks denote significant differences between tree samples and blanks (*P* < 0.05; Wilcoxon test). No significant differences among tree species were detected in panels b, c, or d

Ammonium (NH_4_^+^), nitrite (NO_2_^−^), and nitrate (NO_3_^−^) contents were quantified in shoots and wood cores across all trees (Fig. 2) and corresponding site-specific data are presented in Fig. S1a and S1b. In shoots, NH_4_⁺ content ranged from 1.1 to 5.0 µg g⁻¹ FW across tree species. No significant differences were detected among birch, hornbeam and spruce shoot (Fig. 2a). However, Birch had significantly higher NH_4_^+^ levels than beech (effect size = 0.36, 95% CI:0.26–0.50, FDR-adjusted *P* < 0.0001) (Fig. 2a; Table S1). NO_2_^−^ content was significantly higher in spruce (2.0–3.3 µg g⁻¹ FW) compared to beech (effect size = 0.62, 95% CI: 0.43–0.87, FDR-adjusted *P* = 0.0193) and birch (effect size = 0.44, 95% CI: 0.33–0.59, FDR-adjusted *P* < 0.0001), while hornbeam did not differ significantly. Shoot NO_3_^−^ content was significantly higher in birch (0.9–3.4 µg g⁻¹ FW) than in beech (effect size = 0.51, 95% CI: 0.35–0.73, FDR-adjusted *P* = 0.0005), hornbeam (effect size = 2.69, 95% CI: 1.74–4.16, FDR-adjusted *P* < 0.0001), and spruce (effect size = 2.85, 95% CI:1.88–4.31, FDR-adjusted *P* < 0.0001). In wood cores, no significant interspecific differences were detected for NH_4_^+^, NO_2_^−^, or NO_3_^−^ (Fig. 2b; Table S2). Across tissues, NH_4_^+^ was significantly lower in shoots than wood cores (*F* = 34.85, *P* < 0.001), whereas NO_2_^−^ was higher in shoots (*F* = 22.29, *P* < 0.001), but NO_3_^−^ did not differ significantly between tissues (Table 2).

**Table 2:** Significant differences between tree species (hornbeam, beech, birch, spruce), tree-tissues (shoot, wood cores) and their interactions for each gene and nutrient based on non-parametric Aligned Rank Transform ANOVA

	DF.res	Tree tissues		Tree species		Tree tissues*Tree species	
		F value	*P* value	F value	*P* value	F value	*P* value
*nirK*	43	15.12	< 0.001	2.80	0.05	17.21	< 0.001
*nirS*	43	14.24	< 0.001	3.36	< 0.05	16.86	< 0.001
*norB*	43	8.86	< 0.01	1.59	0.20	19.30	< 0.001
*nosZ* clade I	43	10.69	< 0.01	3.96	< 0.05	8.17	< 0.001
*nosZ* clade II	43	13.14	< 0.001	3.67	< 0.05	6.47	< 0.01
Bacterial *amoA*	43	20.75	< 0.001	3.13	< 0.05	13.94	< 0.001
Archaeal *amoA*	43	22.07	< 0.001	3.02	< 0.05	5.03	< 0.01
*nxrB*	43	14.34	< 0.001	3.83	< 0.05	11.70	< 0.001
Ammonia	41	34.85	< 0.001	1.79	0.16	1.64	0.19
Nitrite	41	22.29	< 0.001	7.72	< 0.001	0.67	0.57
Nitrate	41	0.03	0.87	0.80	0.50	4.44	< 0.01

To account for species-level differences confounded with sampling location, we fitted generalized linear mixed-effects models including location as a random effect (Table S3). In shoots, species effects explained 70–75% of the variance (marginal R^2^), increasing to 72–77% when the location random effects were included (conditional R^2^). This small increase indicates that this minimal increase in the observed species differences was not substantially influenced by location. In wood cores, species effects explained 9–11% of the variance (marginal R^2^). The random effect of location was singular (variance estimated at or near zero), and therefore conditional R² was not computed, suggesting minimal influence of sampling location on nutrient variability.

### N_2_O Exchanges During the Incubation of Shoots

The N_2_O exchange rate during the incubation of shoots indicated varying potential for N_2_O production and consumption depending on tree species (Fig. 2c), with site-specific data provided in supplementary (Fig. S2a). Hornbeam shoots exhibited a positive N_2_O exchange rate (0.002–0.007 ng N_2_O g⁻¹ h⁻¹), suggesting N_2_O emission whereas beech showed a negative exchange rate (− 0.001 to − 0.017 ng N_2_O g⁻¹ h⁻¹), suggesting N_2_O consumption. Both species exhibited N_2_O exchange rates that were significantly (*P* < 0.05) different from the blank controls. We also measured CO_2_ concentration during the incubations to check if the shoots were alive and respiring, and both beech and spruce showed consumption (Fig. S2b). Linear mixed effect model revealed that tree species effects explained 40% of the variance (marginal R^2^), increasing to 59% when location random effects were included (conditional R^2^). Together, these results highlight the combined influence of species identity and location on variability in the N_2_O exchanges.

### N_2_O Concentrations in Wood Bore Holes

The internal N_2_O concentration in relation to ambient air ($\:\varDelta\:$ N_2_O concentration) in wood bore holes varied across locations (Fig. S3). Negative value (e.g., − 9.74 ppb in beech) does not indicate a negative gas concentration but reflects the difference between the hole and ambient concentrations, representing potential N_2_O consumption by the wood (Fig. 2d). The mean Δ N_2_O concentration of hornbeam and spruce indicated potential N_2_O consumption, whereas in birch suggested potential N_2_O emission. A linear mixed-effects model revealed that tree species effects explained 29% of the variance (marginal R²). Including location as a random effect increased the explained variance to 79% (conditional R²), indicating that spatial variation accounted for a large proportion of total variance.

### Relative Abundance of Genes Indicating Genetic Potential for N_2_O Metabolism

**Fig. 3:**
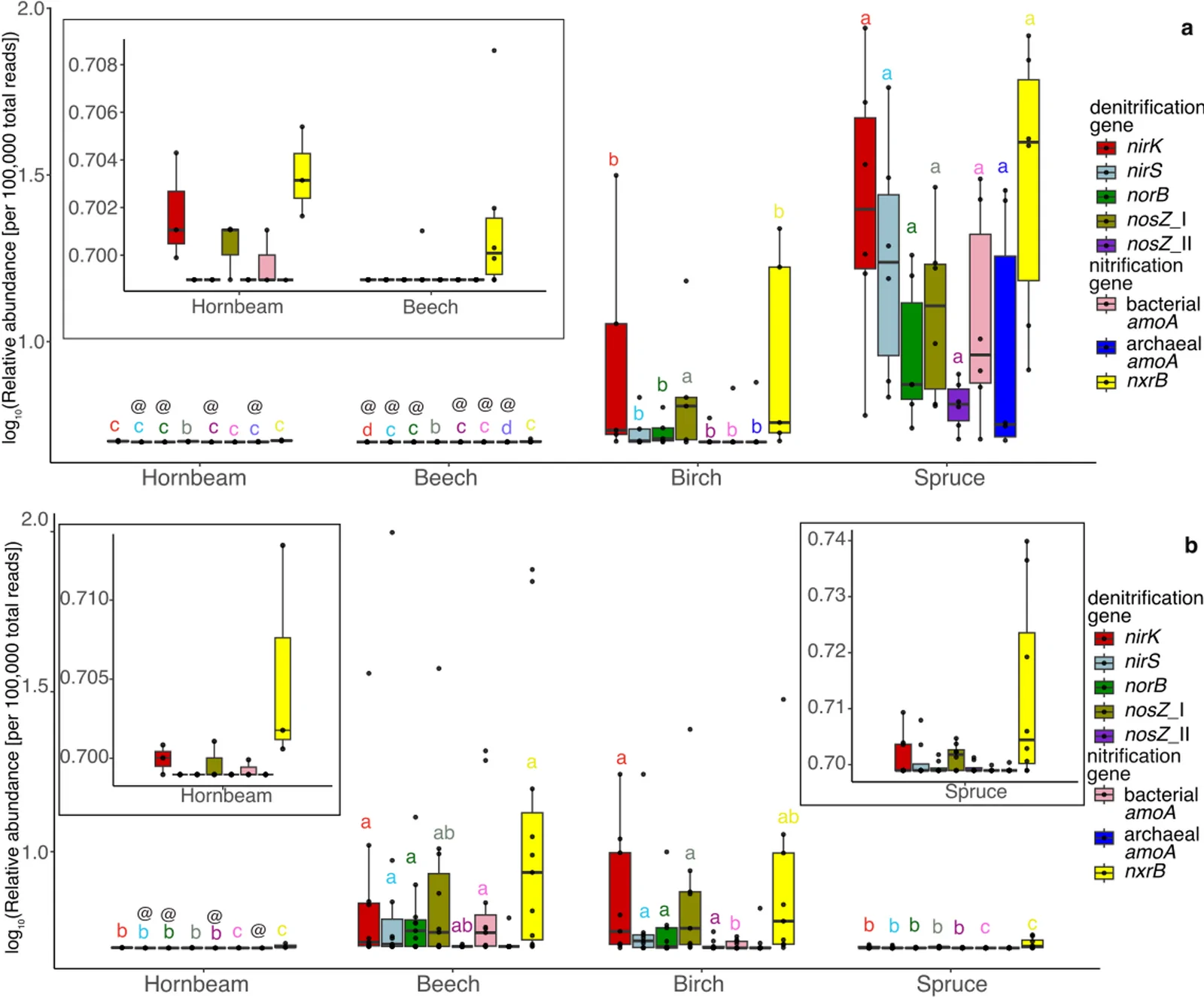
Relative abundances of denitrification and nitrification genes in (**a**) shoots and (**b**) wood cores from different tree species. Relative abundances were calculated as total counts in relation to total reads in post-hybridization libraries. The data were log-transformed for better visualization. The box plots showed all the data points as black dots, and the black line inside the box represented the median (*n* ≥ 3). Significant differences were indicated by different letters and were determined separately for each gene across tree species in panels a and b (FDR-adjusted *P* < 0.05; pairwise comparison of estimated marginal means; Table S1 and S2). Tree species where no reads of N-cycling genes were marked with the symbol @. *nosZ* clade II and archaeal *amoA* in panel b showed no significant difference among tree species

The relative abundance of N-cycling genes varied significantly among tree species in both shoots and wood cores (Fig. 3; corresponding site-specific data are shown in Figs. S4–S7). In shoots, *nirK* dominated and *nirK* abundance was significantly higher in spruce than birch (effect size = 0.25, 95% CI: 0.07–0.95, FDR-adjusted *P* = 0.041; Fig. 3a; Table S1), hornbeam (effect-size = 0.003, 95% CI:0–0.061, FDR-adjusted *P* < 0.001), and beech (effect-size = 0.0001, 95% CI:0–0.001, FDR-adjusted *P* < 0.0001). Among nitrification genes, *nxrB* dominated and spruce had significantly higher *nxrB* abundance than birch (effect-size = 0.17, 95% CI: 0.04–0.73, FDR-adjusted *P* < 0.001), hornbeam (effect-size = 0.003, 95% CI:0–0.058, FDR-adjusted *P* = 0.0002), and beech (effect-size = 0.001, 95% CI:0–0.012, FDR-adjusted *P* < 0.0001).

In wood cores, *norB* dominated and Beech had significantly higher *norB* abundance than spruce (effect-size = 175.46, 95% CI:39.04–788.63, FDR-adjusted *P* < 0.0001; Fig. 3b; Table S2) and hornbeam (effect-size = 1307.54, 95% CI:159.05–10,748.90, FDR-adjusted *P* < 0.0001) with no significant difference compared to birch. For nitrification genes in wood cores, Beech showed significantly higher *nxrB* abundance than hornbeam (effect-size = 72.77, 95% CI:2.33–2269.80, FDR-adjusted *P* = 0.0292) and spruce (effect-size = 85.36, 95% CI:7.09–1027.68, FDR-adjusted *P* = 0.0014), with no significant difference compared to birch.

The gene coding for enzyme responsible for reducing N_2_O to N_2_, *nosZ*, showed a strong dominance of clade I over clade II in both shoot and wood cores. In shoots, spruce had significantly higher *nosZ* clade I abundance than hornbeam (effect size = 0.002, 95% CI: 0–0.012, FDR-adjusted *P* < 0.0001; Table S1) and beech (effect size = 0.001, 0–0.012, FDR-adjusted *P* < 0.0001) with no significant difference compared to birch. In wood cores, birch had significantly higher *nosZ* clade I abundance than spruce (effect-size = 92.25, 95% CI:19.13–444.89, FDR-adjusted *P* < 0.0001; Table S2) and hornbeam (effect-size = 227.66, 95% CI:3.57–14,533.65, FDR-adjusted *P* = 0.03), with no significant difference with beech.

Generalized linear mixed-effects models showed that most N-cycling genes in shoots had location-level random-effect variances below 10%, indicating that tree-species differences were generally not driven by sampling location. The exception was archaeal *amoA*, whose variance increased from 72% to 95% when location was included. In wood cores, tree species explained 85–88% of the variance for *norB* and bacterial *amoA*, and the location effect was singular, indicating minimal location influence. However, for other genes in wood cores tree species were confounded with location, suggesting that spatial variation local factors contributed to the observed species patterns.

### Diversity and Distribution of ***nosZ*** Genes Among the Trees

**Fig. 4:**
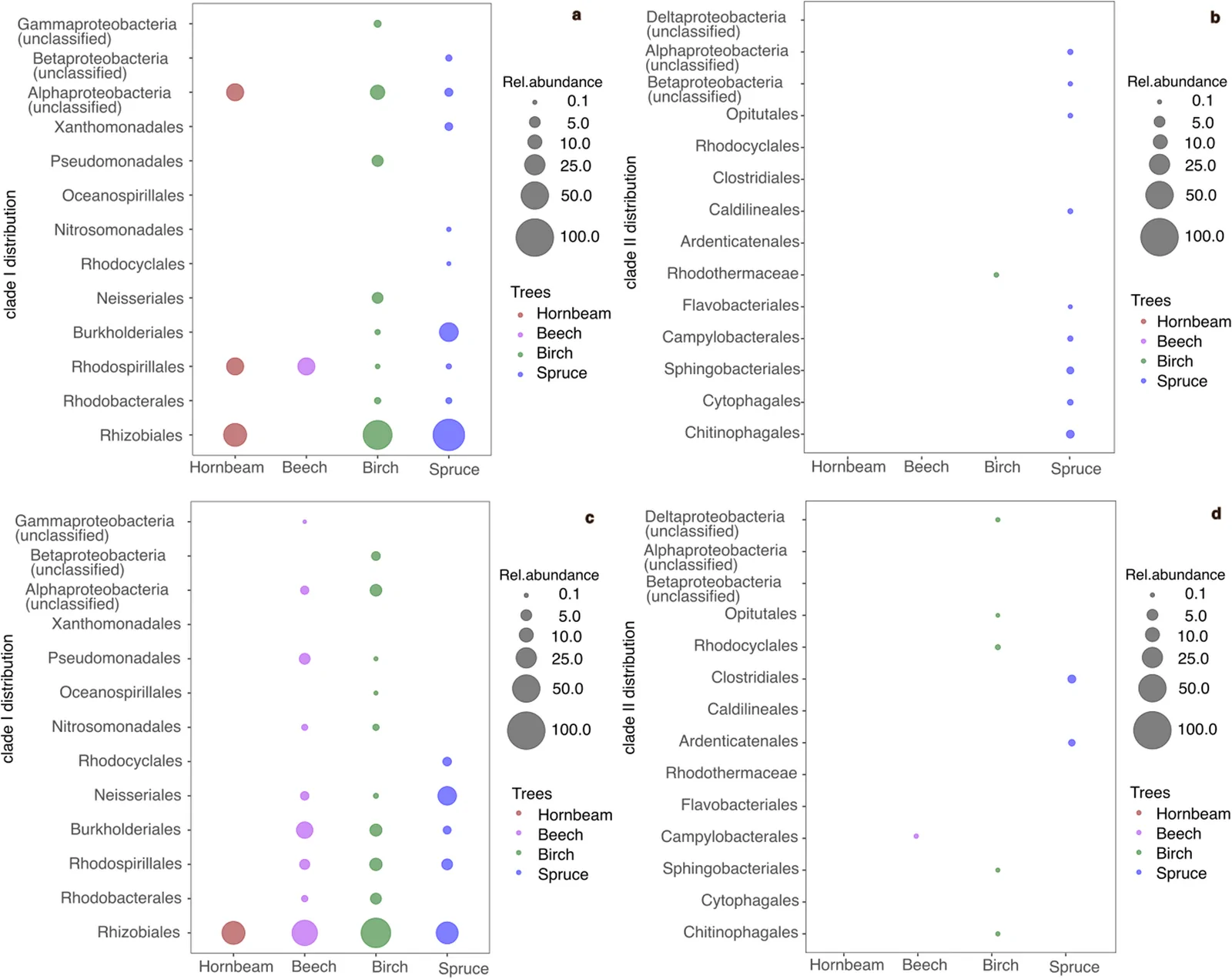
*nosZ* clade I and II distribution based on the taxonomic classification at the order level in shoots (**a** and **b**) and in wood cores (**c** and **d**)

In shoot tissues, *nosZ* clade I genes were predominantly associated with *nosZ* in Alphaproteobacteria, particularly *Rhizobiales* and *Rhodospirillales* (Fig. 4a). A higher diversity of *nosZ* clade I community was observed in spruce and birch shoots, which mainly included *Rhizobiales*, *Rhodobacterales*, *Rhodospirillales*, and *Burkholderiales.* Higher diversity of *nosZ* clade II communities was noted in spruce trees, which included *Chitinophagales*, *Cytophagales*, *Sphingobacteriales*, *Campylobacterales*, *Flavobacteriales*, *Caldilineales*, and *Opitutales* (Fig. 4b). Notably, *nosZ* clade II communities were below detection limit in hornbeam and beech shoots, and *Rhodothermaceae* showed very low abundance in birch. Wood cores displayed high diversity of *nosZ* clade I communities in beech and birch (Fig. 4c) and shared taxa between these trees included *Rhizobiales*, *Rhodobacterales*, *Rhodospirillales*, *Burkholderiales*, *Neisseriales*, *Nitrosomonadales*, and *Pseudomonadales*. Birch wood cores also exhibited diverse *nosZ* clade II communities, comprising *Chitinophagales*, *Sphingobacteriales*, *Rhodocyclales*, and *Opitutales* (Fig. 4d). No *nosZ* clade II communities were detected in hornbeam wood cores.

### Linkages Between Nutrient Contents, Activity, and Relative Abundance of N-Cycling Microorganisms

The study demonstrated significant variation in both N-cycling genes and nutrient contents (NH_4_^+^, NO_2_^−^) across tree tissues, indicating tissue-specific distribution (Table 2). Additionally, the relative abundance of key N-cycling genes (*nirK*, *nirS*, *nosZ* clades I and II, bacterial and archaeal *amoA*, *nxrB*) and NO_2_^−^ content differed significantly among tree species, suggesting tree-specific patterns (Table 2). Importantly, a strong interaction between tree species (hornbeam, beech, birch, spruce) and tissues (shoots and wood cores) influenced all N-cycling genes and NO_3_^−^ content, underscoring that tree species–tree tissue relationships jointly shape N-cycling dynamics.

**Fig. 5:**
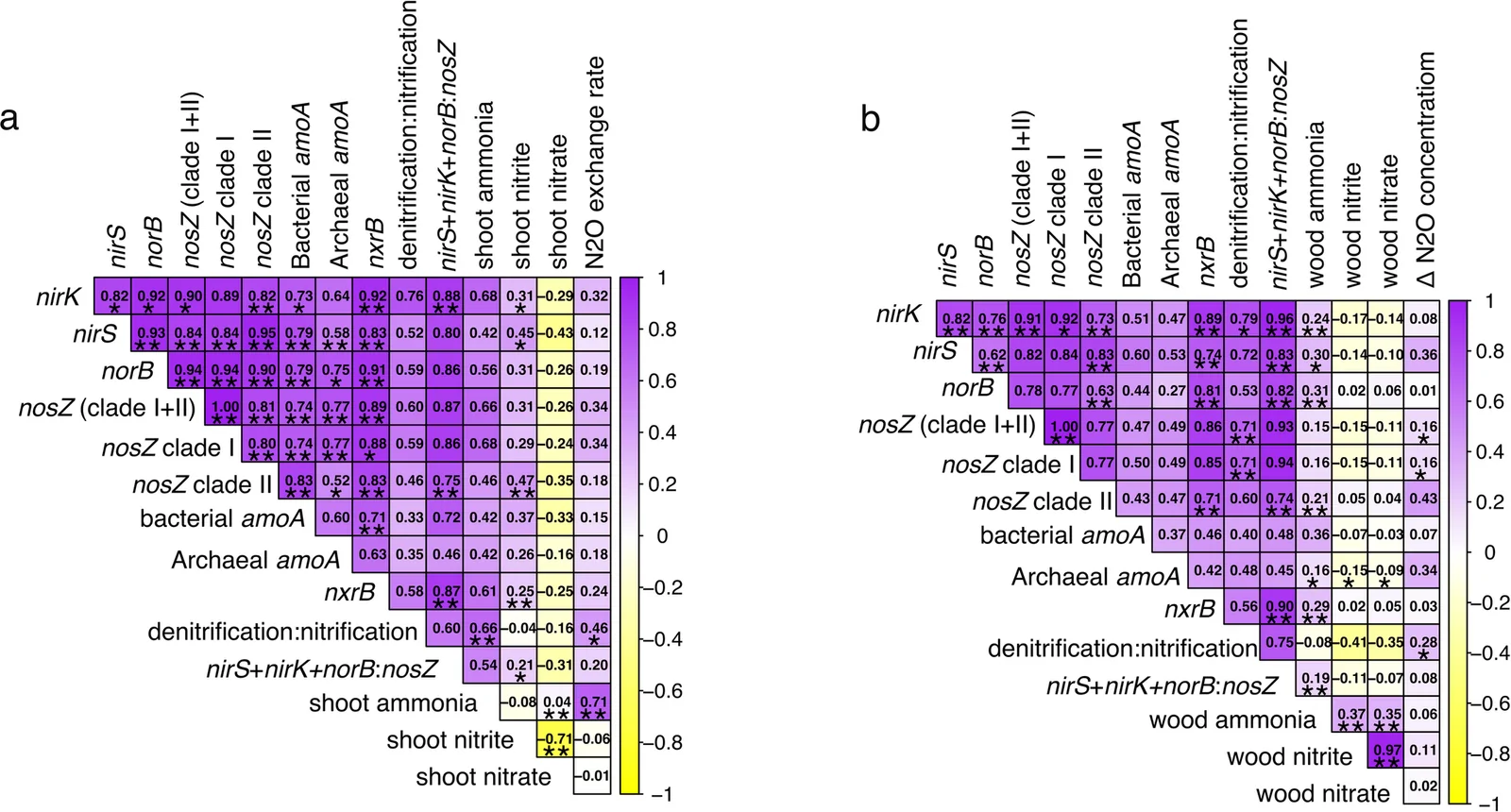
Correlation analysis for environmental and microbial variables of the shoot (**a**) and wood cores (**b**). The color intensity showed the Spearman correlation coefficient and the color indicates the direction. Asterisk represented a significant difference of 0.01 (**) and 0.05 (*) between parameters

In shoots, NH_4_^+^ content was significantly positively associated with denitrification: nitrification gene abundance ratio, while NO_2_^−^ content showed significant positive correlations with *nirK*, *nirS*, *nosZ* clade II, *nxrB*, (*nirS*+*nirK*+*norB*):*nosZ* ratio (Fig. 5a; corresponding ratio data are shown in Fig. S8). The N_2_O exchange rate was also significantly correlated with both the denitrification: nitrification gene ratio and shoot NH_4_^+^ content. In wood cores, NH_4_^+^ content exhibited significant positive correlations with *nirK*, *nirS*, *norB*, *nosZ* clade II, archaeal *amoA*, *nxrB*, (*nirS*+*nirK*+*norB*):*nosZ* ratio (Fig. 5b). Furthermore, Δ N_2_O concentration was significantly associated with *nosZ* clade I and the denitrification: nitrification gene ratio. A comparable relationship was observed in linear models assessing the linkage between N_2_O exchange, nutrient content, and the relative abundance of N-cycling genes (Table 3).

**Table 3:** Relationship between N_2_O exchanges, nutrient content and the relative abundance of N-cycling genes based on step-wise linear models

Model	Selected models	AIC Value	F value	*P* value
Shoot N_2_O exchange ~ shoot ammonia + shoot nitrite + shoot nitrate	Shoot ammonia	−208.85	11.98	< 0.001
Shoot N_2_O exchange ~ *nirK* + *nirS* + *norB* + *nxrB* + archaeal *amoA* + bacterial *amoA* + *nosZ* + *nosZ* clade I + *nosZ* clade II + denitrification: nitrification ratio + (*nirS*+*nirK+norB*):*nosZ* ratio	denitrification: nitrification gene ratio	−205.18	6.96	< 0.05
Wood N_2_O concentration ~ *nirK* + *nirS* + *norB* + *nxrB* + archaeal *amoA* + bacterial *amoA* + *nosZ* + *nosZ* clade I + *nosZ* clade II + denitrification: nitrification gene ratio + (*nirS*+*nirK+norB*):*nosZ* ratio	*nosZ* *nosZ* clade I denitrification: nitrification gene ratio	−57.60 −57.53 −56.52	7.51 7.42 6.23	< 0.05 < 0.05 < 0.05

## Discussion

### Prevalence of N-Cycling Communities and Nutrient Content in Tree Tissues

Both shoots and wood cores contained genes present in nitrifying and denitrifying microorganisms and inorganic N species, indicating these tissues are potential active sites for microbial N transformations and N_2_O exchange. Spruce and birch shoots exhibited higher relative abundances of these genes compared to other trees, while birch and beech wood cores showed elevated levels, indicating tissue-specific microbial colonization. Internal tree tissues create unique microhabitats for N-cycling microbes because gases diffuse slowly, leading to microaerophilic zones [15, 42]. For example, sapwood and heartwood harbor distinct microbial communities, including anaerobic taxa, showing that internal wood environments can support processes normally associated with low‑oxygen conditions alongside aerobic pathways when oxygen is available [15]. Leaves also form distinct niches: phyllosphere and endophytic microbes [43] experience strong fluctuations in humidity, temperature, and atmospheric inputs, selecting for metabolically flexible microbial communities [44]. These combined structural and environmental features explain why microbes living inside leaves and wood often have functional capacities that differ from those found in soils or the rhizosphere.

Shoots exhibited significantly higher NO_2_^-^ concentrations than wood cores, likely because photosynthetic tissues actively assimilate NO_3_^-^. During this process, NO_3_^-^ is first reduced to NO_2_^-^ in the cytosol before further reduction to NH_4_^+^ in chloroplasts, and the intermediate NO_2_^-^ can accumulate in shoots [45]. Leaves also have easier access to atmospheric NO_3_^-^ via stomatal uptake of nitrogen dioxide (NO_2_), providing a leaf‑specific supplementary pathway that is not available to wood cores [46]. By contrast, wood cores contained higher NH_4_⁺ concentrations, indicating potential nitrification within these tissues. The nutrient patterns correlated with relative abundance of N-cycling genes and N_2_O exchange. Elevated NO_2_^-^ level in shoots was associated with higher *nxrB* relative abundance, suggesting that nitrite‑oxidizing bacteria are present in NO_2_^-^ rich tissues. Positive correlation was also observed between shoot NO_2_^-^ concentrations and *nirK* relative abundance, consistent with findings that elevated NO_2_^-^ can stimulate denitrifying microbial activity and increase *nirK* abundance [47]. In wood cores, the positive correlation between NH_4_^+^ and several N-cycling genes (*nirK*, *nirS*, *norB* and *nxrB*) suggests that NH_4_^+^-rich wood environments support microbial communities containing both nitrifiers and denitrifiers.

### N_2_O Reducing Microorganisms

Diverse *nosZ* clade I organisms dominated over *nosZ* clade types in both shoots (birch and spruce) and wood cores (beech, birch and spruce). Alphaproteobacteria with *Rhizobiales* orders were notably dominant, and members from this order alongside taxa from *Burkholderiales*, *Neisseriales*, and *Rhodobacterales*, have recently been confirmed in spruce shoots [13]. Other clade I organisms, including *Oceanospillales* (uniquely present in wood cores) and *Xanthomonadales* (uniquely present in shoots), indicate tissue-specific niches for specialized N_2_O-transforming microbes. Although clade II organisms were less abundant, they comprised a more diverse set of taxa in spruce shoots and birch wood cores, suggesting that even low-abundance taxa may be important for N_2_O reduction. Some common communities detected in shoot and wood cores include *Chitinophagales*, *Sphingobacteriales*, *Campylobacterales*, and *Opitutales*, previously reported in soils, lakes, and wastewater systems as denitrifiers [48–50].

In contrast to the observed dominance of *nosZ* clade I N_2_O reducers in tree tissues, *nosZ* clade II is often dominant in soil and lake systems [17] and the higher diversity or abundance of *nosZ* clade II is typically linked to lower net N_2_O emissions or increased N_2_O consumption [51, 52]. Tree tissues experience micro‑oxic to anoxic microzones and redox oscillations, which favor complete denitrifiers [53], often carrying clade I *nosZ* [54], that can maintain denitrification readiness and rapidly reduce N_2_O when O_2_ drops [55]. Clade I dominated N_2_O reducing communities have been reported in root microbiomes [54] and spruce-associated microbiome [13], indicating plant tissues may selectively favor clade I taxa. This pattern was evident across all trees but was particularly pronounced in spruce shoots and beech wood cores, which showed high abundance and diversity of *nosZ* clade I. The difference in *nosZ* communities detected across tree species aligns with previous studies showing that leaf traits affect fungal communities in trees [56]. Together, these findings highlight the presence of diverse N_2_O-reducing communities in aboveground tree tissues.

### N_2_O Exchange Pattern Across Trees

Shoot tissues could either emit or consume N_2_O, with beech shoots notably exhibiting negative exchange rates, indicative of N_2_O consumption, and hornbeam showing positive N_2_O exchange rates, suggesting potential for N_2_O emission. Wood core gas concentrations further supported these findings. Interestingly, in Agali, birch stems showed net N_2_O consumption, whereas the corresponding internal wood cores exhibited high N_2_O emission. This pattern suggests that the high abundance of *nosZ*‑carrying microbes in the wood may have reduced a substantial fraction of internally produced N_2_O before it could diffuse to the atmosphere. The low relative abundances of *nosZ* clade I in beech does not explain the sink capacity of beech, although low relative abundance does not necessarily indicate low activity. Future studies should address this by quantifying absolute gene counts through qPCR and activity using metatranscriptomics. Further, although the differences between species suggest species‑specific microbial activity, variation in bore‑hole depth, stem diameter, and the physiological age of sampled wood tissues, as well as the fact that shoot sampling height could not be fully standardised and species differ in crown structure and light exposure that shape shoot physiology, may also contribute to the observed patterns.

Nevertheless, our results align with studies reporting beech stems (Štítná nad Vláří, Czech Republic; [22]) and beech shoots (Freising, Germany; [8]) acting as atmospheric N_2_O sinks, and N_2_O emissions from birch in Estonia [7]. Other studies have also reported N_2_O fluxes from tree stems and shoots, including spruce and birch stems [11], and Scots pine shoots and stems as N_2_O sources [10]. Variability in emissions across trees underscores the influence of environmental and physiological factors [4, 10, 57]. The abundance of lenticels in birch may have contributed to higher N_2_O emissions from stem wood, as birch possesses a greater density of lenticels compared to other species. This anatomical feature can facilitate bark-mediated gas diffusion [58]. Additionally, stem N_2_O emissions have been linked to soil N_2_O fluxes, especially when stem chambers are positioned near the soil surface [11, 59]. This suggests that some observed in situ fluxes may result from upward transport of soil-derived N_2_O. Recent work also suggests that N_2_O fixation may serve as a biological sink in aquatic environments [60], but our data do not allow us to determine whether this process occurs in tree tissues.

## Conclusion

This study demonstrates that shoot and wood cores tissues harbour microbial communities involved in nitrification, denitrification and N_2_O reduction and may serve as active sites of microbial N-cycling. We observed differences among tree species and tree tissues, but because species were sampled where they naturally dominate, species and site effects inevitably overlap. Including location as a random factor mitigates but cannot fully resolve this. Despite these challenges, the consistent alignment between nutrient patterns, microbial gene profiles, and gas-exchange signals demonstrates that microbial processes within tree tissues likely contribute to N_2_O dynamics. Our findings further suggest that internal nutrient availability, especially NH_4_^+^ and NO_2_^−^, plays a significant role in shaping microbial community structure and function. These insights are relevant for forest N_2_O budgets and biogeochemical feedback. Incorporating tree‑associated microbial processes into ecosystem‑scale assessments may improve predictions of forest N_2_O exchange, particularly because current models only consider soil sources. Future research integrating functional assays, isotopic approaches, and in situ flux measurements will be essential to quantify microbial contributions and to clarify the mechanisms driving N_2_O fluxes in forest ecosystems, which may serve as active sites of microbial N_2_O metabolism.

## Supplementary Information

Below is the link to the electronic supplementary material.

Supplementary Material 1 (DOCX 5.32 MB)

## Data Availability

The metagenomic sequencing data in this paper have been deposited at the SRA-NCBI (https://www.ncbi.nlm.nih.gov/) under BioProject accession no.: PRJNA1307397 ([https://www.ncbi.nlm.nih.gov/bioproject/1307397](https:/www.ncbi.nlm.nih.gov/bioproject/1307397)).
