# Supplementary material for: Genetic Potential for N₂O Metabolism in Tree Tissues: Insights From Nitrogen Cycling Gene Prevalence and nosZ Diversity Across Tree Species
Source: Microb Ecol. 2026 Apr 18;89(1):116. doi: 10.1007/s00248-026-02773-8 (PMC13219165; doi:10.1007/s00248-026-02773-8)
Supplement: Supplementary file 1 — Supplementary Material 1 (DOCX 5.32 MB) [file 248_2026_2773_MOESM1_ESM.docx]

**Supplementary material**

**Genetic potential for N₂O metabolism in tree tissues: Insights from nitrogen cycling gene prevalence and *nosZ* diversity across tree species**

Krishnapriya Thiyagarasaiyar^1^, Dhiraj Paul^1^, Johanna Kerttula^1^, Milja Keski-Karhu^1^, Kaido Soosaar^2^, Ülo Mander^2^, Sara Hallin^3^, Katerina Machacova^4^, Jukka Pumpanen^1^, Henri M.P. Siljanen^1^

^1^ Department of Environmental and Biological Sciences, University of Eastern Finland, Kuopio, Finland

^2^ Department of Geography, Tartu University, Tartu, Estonia

^3^ Department of Forest Mycology and Plant Pathology, Swedish University of Agricultural Sciences, Uppsala, Sweden

^4^ Department of Ecosystem Trace Gas Exchange, Global Change Research Institute of the Czech Academy of Sciences, Brno, Czech Republic

*Correspondence:

Email: [krishnapriya.thiyagarasaiyar@uef.fi](mailto:krishnapriya.thiyagarasaiyar@uef.fi)


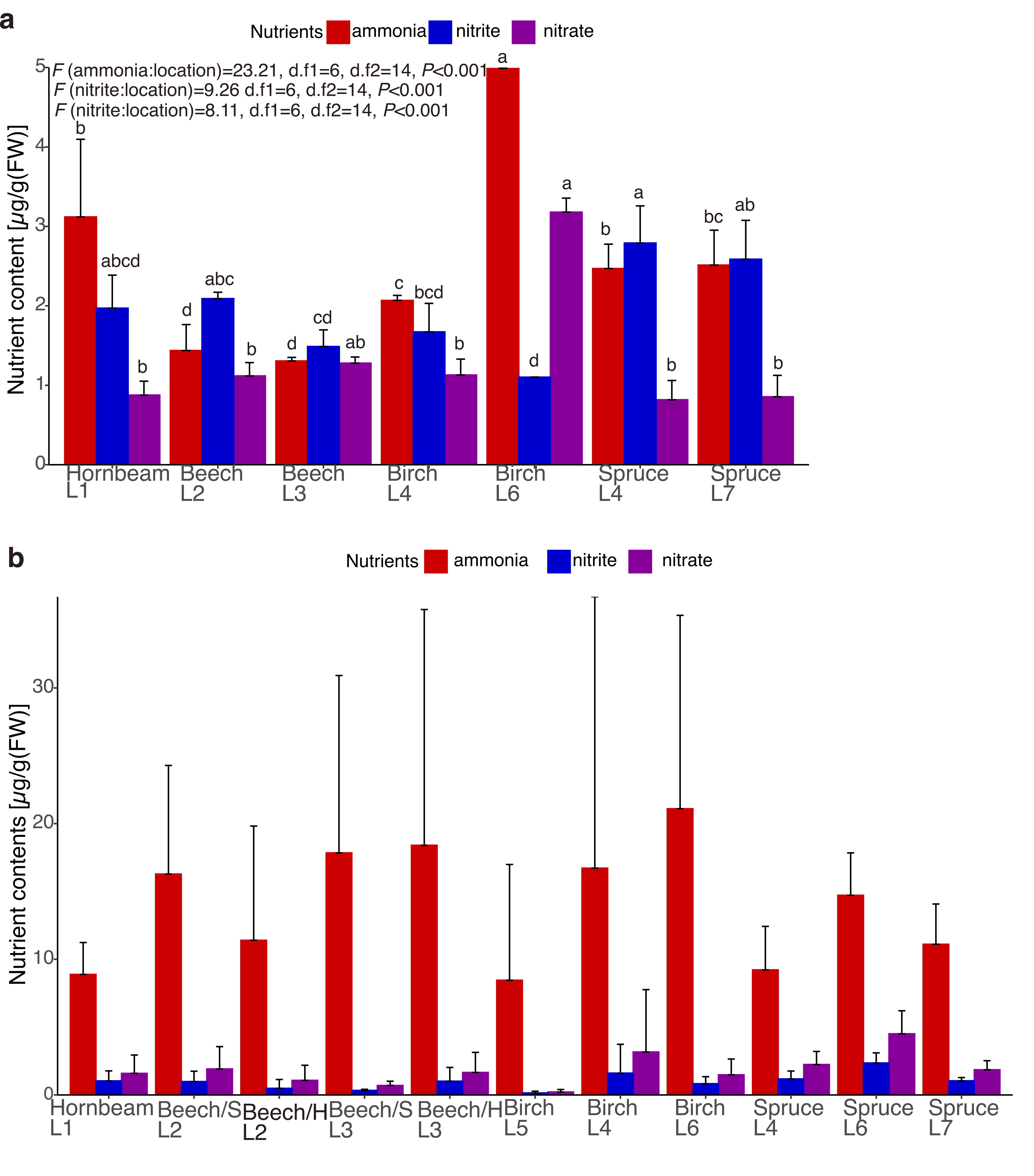


Figure S1: Nutrient content in plant tissues collected from different locations. (a): NH_4_^+^, NO_2_^–^ and NO_3_^–^ concentrations in shoots (mean+standard deviation), (b): NH_4_^+^, NO_2_^–^ and NO_3_^–^ in wood cores (mean+standard deviation). Significant differences (*P* < 0.05; dunn’s test with bonferroni correction) are indicated by different letters and were determined separately for each nutrients across locations in (a). No significant differences were observed in (b). Beech/S means beech sapwood and Beech/H means beech heartwood in b. L1= Lanžhot, Czech Republic; L2= Štítná nad Vláří, Czech Republic; L3= Bromarv, Finland; L4=Agali, Estonia; L5= Kiidjärve, Estonia; L6=Puijo, Finland; L7=Kenttärova-Pallas, Finland.

Table S1. Pairwise species comparisons of N‑cycling gene abundance, nutrient contents and N_2_O exchanges in shoots.

| Analysis | Comparison | Effect size | 95% CI | p.value | p. value (FDR.adj) |
| --- | --- | --- | --- | --- | --- |
| Ammonia | Beech / Birch | 0.36 | 0.26–0.50 | <0.0001 | <0.0001 |
|  | Beech/hornbeam | 0.44 | 0.30–0.65 | <0.0001 | <0.0001 |
|  | Beech/Spruce | 0.56 | 0.39–0.81 | 0.0018 | 0.0037 |
|  | Birch / hornbeam | 1.23 | 0.83–1.82 | 0.3099 | 0.3099 |
|  | Birch / Spruce | 1.57 | 1.09–2.28 | 0.0161 | 0.0241 |
|  | hornbeam / Spruce | 1.28 | 0.84–1.96 | 0.2480 | 0.2976 |
| Nitrite | Beech / Birch | 1.40 | 0.98–2.00 | 0.0614 | 0.0921 |
|  | Beech/hornbeam | 0.89 | 0.58–1.37 | 0.6006 | 0.6006 |
|  | Beech/Spruce | 0.62 | 0.43–0.87 | 0.0064 | 0.0193 |
|  | Birch / hornbeam | 0.64 | 0.41–0.98 | 0.0406 | 0.0812 |
|  | Birch / Spruce | 0.44 | 0.33–0.59 | <0.0001 | <0.0001 |
|  | hornbeam / Spruce | 0.69 | 0.45–1.06 | 0.0866 | 0.1039 |
| Nitrate | Beech / Birch | 0.51 | 0.35–0.73 | 0.0002 | 0.0005 |
|  | Beech / hornbeam | 1.37 | 0.89–2.09 | 0.1495 | 0.1794 |
|  | Beech / Spruce | 1.45 | 0.97–2.16 | 0.0721 | 0.1082 |
|  | Birch / hornbeam | 2.69 | 1.74–4.16 | <0.0001 | <0.0001 |
|  | Birch / Spruce | 2.85 | 1.88–4.31 | <0.0001 | <0.0001 |
|  | hornbeam / Spruce | 1.06 | 0.66–1.70 | 0.8139 | 0.8139 |
| N_2_O exchange | Birch/Beech | -1.58 | -0.02-0.01 | 0.2876 | 0.5751 |
|  | Hornbeam/Beech | -2.75 | -0.03-0.01 | 0.1829 | 0.5751 |
|  | Spruce/Beech | -1.60 | -0.02-0.01 | 0.2817 | 0.5751 |
|  | Hornbeam/Birch | -1.17 | -0.03-0.02 | 0.5017 | 0.6027 |
|  | Birch / Spruce | -0.01 | -0.01-0.01 | 0.9910 | 0.9910 |
|  | hornbeam / Spruce | 1.06 | -0.02 -0.03 | 0.5022 | 0.6027 |
| *nirK* | Beech/Birch | 0.0004 | 0.000–0.004 | <0.0001 | <0.0001 |
|  | Beech/Hornbeam | 0.03 | 0.001–0.62 | 0.0235 | 0.0282 |
|  | Beech/Spruce | 0.0001 | 0–0.001 | <0.0001 | <0.0001 |
|  | Birch / hornbeam | 77.68 | 4.19–1440.17 | 0.0035 | 0.0052 |
|  | Birch / Spruce | 0.25 | 0.07–0.95 | 0.0411 | 0.0411 |
|  | hornbeam / Spruce | 0.003 | 0–0.061 | 0.0001 | 0.0003 |
| *nirS* | Beech/Birch | 0.002 | 0.001–0.006 | <0.0001 | <0.0001 |
|  | Beech / hornbeam | 1.00 | 0.28–3.57 | 1 | 1 |
|  | Beech/Spruce | 0.0001 | 0–0 | <0.0001 | <0.0001 |
|  | Birch / hornbeam | 463.89 | 124.531–1728.038 | <0.0001 | <0.0001 |
|  | Birch/Spruce | 0.03 | 0.009–0.077 | <0.0001 | <0.0001 |
|  | Hornbeam/Spruce | 0.0001 | 0–0 | <0.0001 | <0.0001 |
| *norB* | Beech/Birch | 0.002 | 0.001–0.007 | <0.0001 | <0.0001 |
|  | Beech / hornbeam | 1.00 | 0.322–3.106 | 1 | 1 |
|  | Beech/Spruce | 0.0002 | 0–0 | <0.0001 | <0.0001 |
|  | Birch / hornbeam | 405.27 | 125.70–1306.57 | <0.0001 | <0.0001 |
|  | Birch/Spruce | 0.08 | 0.03–0.208 | <0.0001 | <0.0001 |
|  | Hornbeam/Spruce | 0.0002 | 0–0.001 | <0.0001 | <0.0001 |
| *nosZ* clade I | Beech/Birch | 0.002 | 0–0.009 | <0.0001 | <0.0001 |
|  | Beech/Hornbeam | 0.29 | 0.05–1.79 | 0.1807 | 0.1807 |
|  | Beech/Spruce | 0.001 | 0–0.002 | <0.0001 | <0.0001 |
|  | Birch / hornbeam | 156.86 | 23.76–1035.52 | <0.0001 | <0.0001 |
|  | Birch/Spruce | 0.29 | 0.06–1.39 | 0.1221 | 0.1465 |
|  | Hornbeam/Spruce | 0.002 | 0–0.012 | <0.0001 | <0.0001 |
| *nosZ* clade II | Beech/Birch | 0.02 | 0.002–0.15 | 0.0003 | 0.0006 |
|  | Beech / hornbeam | 1.00 | 0.06–16.87 | 1.0000 | 1.0000 |
|  | Beech/Spruce | 0.002 | 0–0.017 | <0.0001 | <0.0001 |
|  | Birch / hornbeam | 61.59 | 3.90–973.05 | 0.0034 | 0.0041 |
|  | Birch/Spruce | 0.11 | 0.03–0.47 | 0.0031 | 0.0041 |
|  | Hornbeam/Spruce | 0.002 | 0–0.029 | <0.0001 | <0.0001 |
| Bacterial *amoA* | Beech/Birch | 0.002 | 0.001–0.01 | <0.0001 | <0.0001 |
|  | Beech/Hornbeam | 0.11 | 0.02–0.63 | 0.0128 | 0.0128 |
|  | Beech/Spruce | 0.0001 | 0–0 | <0.0001 | <0.0001 |
|  | Birch / hornbeam | 50.00 | 8.34–299.66 | <0.0001 | <0.0001 |
|  | Birch/Spruce | 0.05 | 0.011–0.213 | <0.0001 | <0.0001 |
|  | Hornbeam/Spruce | 0.001 | 0–0.005 | <0.0001 | <0.0001 |
| Archaeal *amoA* | Beech/Birch | 0.033 | 0.001–1.308 | 0.0692 | 0.1038 |
|  | Beech / hornbeam | 1.00 | 0.01–118.44 | 1.0000 | 1.0000 |
|  | Beech/Spruce | 0.002 | 0–0.071 | 0.0008 | 0.0046 |
|  | Birch / hornbeam | 30.29 | 0.31–3004.13 | 0.1459 | 0.1751 |
|  | Birch/Spruce | 0.05 | 0.01–0.35 | 0.0024 | 0.0071 |
|  | Hornbeam/Spruce | 0.002 | 0–0.177 | 0.0070 | 0.0140 |
| *nxrB* | Beech/Birch | 0.007 | 0.001–0.066 | <0.0001 | <0.0001 |
|  | Beech/Hornbeam | 0.35 | 0.02–6.51 | 0.4837 | 0.4837 |
|  | Beech/Spruce | 0.001 | 0–0.012 | <0.0001 | <0.0001 |
|  | Birch / hornbeam | 52.50 | 3.11–887.47 | 0.0060 | 0.0091 |
|  | Birch/Spruce | 0.17 | 0.04–0.73 | 0.0168 | 0.0201 |
|  | Hornbeam/Spruce | 0.003 | 0–0.058 | 0.0001 | 0.0002 |

Table S2. Pairwise species comparisons of N‑cycling gene abundance, nutrient contents and N_2_O exchanges in wood cores.

| Analysis | Comparison | Effect size | 95% CI | p.value | p.value (FDR.adj) |
| --- | --- | --- | --- | --- | --- |
| Ammonia | Beech / Birch | 1.01 | 0.58–1.75 | 0.9761 | 0.9761 |
|  | Beech / Hornbeam | 1.75 | 0.80–3.85 | 0.1629 | 0.5242 |
|  | Beech / Spruce | 1.34 | 0.74–2.42 | 0.3290 | 0.5307 |
|  | Birch / Hornbeam | 1.74 | 0.78–3.85 | 0.1747 | 0.5242 |
|  | Birch / Spruce | 1.33 | 0.73–2.43 | 0.3538 | 0.5307 |
|  | Hornbeam/Spruce | 0.77 | 0.34–1.75 | 0.5261 | 0.6313 |
| Nitrite | Beech / Birch | 0.83 | 0.35–1.97 | 0.6728 | 0.7750 |
|  | Beech / Hornbeam | 0.69 | 0.20–2.39 | 0.5591 | 0.7750 |
|  | Beech / Spruce | 0.49 | 0.19–1.25 | 0.1350 | 0.7750 |
|  | Birch / Hornbeam | 0.83 | 0.24–2.93 | 0.7750 | 0.7750 |
|  | Birch / Spruce | 0.59 | 0.23–1.54 | 0.2817 | 0.7750 |
|  | Hornbeam/Spruce | 0.71 | 0.19–2.62 | 0.6100 | 0.7750 |
| Nitrate | Beech / Birch | 0.85 | 0.33–2.16 | 0.7255 | 0.9788 |
|  | Beech / Hornbeam | 0.86 | 0.22–3.31 | 0.8273 | 0.9788 |
|  | Beech / Spruce | 0.50 | 0.18–1.38 | 0.1834 | 0.9163 |
|  | Birch / Hornbeam | 1.02 | 0.26–3.99 | 0.9788 | 0.9788 |
|  | Birch / Spruce | 0.60 | 0.21–1.67 | 0.3267 | 0.9163 |
|  | Hornbeam/Spruce | 0.59 | 0.14–2.41 | 0.4581 | 0.9163 |
| $\Delta$N_2_O concentration | Beech/Birch | -1.81 | -4.04–0.43 | 0.0919 | 0.5518 |
|  | Beech/Hornbeam | -0.57 | -3.57–2.44 | 0.6437 | 0.7724 |
|  | Beech/Spruce | -0.81 | -3.04–1.42 | 0.3925 | 0.5887 |
|  | Birch / Hornbeam | 1.24 | -1.60–4.08 | 0.3055 | 0.5887 |
|  | Birch/Spruce | 0.99 | -1.00–2.99 | 0.2562 | 0.5887 |
|  | Hornbeam/Spruce | -0.25 | -3.08–2.59 | 0.8296 | 0.8296 |
| *nirK* | Beech/Birch | 0.36 | 0.01–10.52 | 0.5508 | 0.6609 |
|  | Beech / Hornbeam | 30.48 | 0.27–3489.34 | 0.1577 | 0.2366 |
|  | Beech / Spruce | 34.46 | 1.16–1020.45 | 0.0406 | 0.0962 |
|  | Birch / Hornbeam | 85.33 | 1.03–7015.82 | 0.0481 | 0.0962 |
|  | Birch / Spruce | 96.49 | 23.92–389.29 | <0.0001 | <0.0001 |
|  | Hornbeam / Spruce | 1.13 | 0.01–93.22 | 0.9565 | 0.9565 |
| *nirS* | Beech / Birch | 3.75 | 0.14–101.58 | 0.4323 | 0.4323 |
|  | Beech / Hornbeam | 1244.27 | 12.99–119221.02 | 0.0022 | 0.0047 |
|  | Beech / Spruce | 168.96 | 6.19–4615.47 | 0.0024 | 0.0047 |
|  | Birch / Hornbeam | 331.79 | 4.53–24279.05 | 0.0080 | 0.0121 |
|  | Birch / Spruce | 45.06 | 7.52–269.96 | <0.0001 | 0,0002 |
|  | Hornbeam / Spruce | 0.136 | 0.002–9.966 | 0.3623 | 0.4323 |
| *norB* | Beech / Birch | 1.69 | 0.394–7.221 | 0.4805 | 0.4805 |
|  | Beech / Hornbeam | 1307.54 | 159.05–10748.90 | <0.0001 | <0.0001 |
|  | Beech / Spruce | 175.46 | 39.04–788.63 | <0.0001 | <0.0001 |
|  | Birch / Hornbeam | 774.78 | 89.69–6692.96 | <0.0001 | <0.0001 |
|  | Birch / Spruce | 103.97 | 21.60–500.55 | <0.0001 | <0.0001 |
|  | Hornbeam/Spruce | 0.13 | 0.02–1.20 | 0.0722 | 0.0867 |
| *nosZ* clade I | Beech/Birch | 0.15 | 0.01–3.53 | 0.2360 | 0.2833 |
|  | Beech / Hornbeam | 33.06 | 0.39–2829.98 | 0.1233 | 0.1850 |
|  | Beech / Spruce | 13.40 | 0.54–330.68 | 0.1127 | 0.1850 |
|  | Birch / Hornbeam | 227.66 | 3.57–14533.65 | 0.0105 | 0.0314 |
|  | Birch / Spruce | 92.25 | 19.13–444.89 | <0.0001 | <0.0001 |
|  | Hornbeam/Spruce | 0.41 | 0.01–26.19 | 0.6710 | 0.6710 |
| *nosZ* clade II | Beech/Birch | 0.10 | 0.01–0.98 | 0.0483 | 0.0966 |
|  | Beech / Hornbeam | 4.58 | 0.20–103.44 | 0.3389 | 0.5083 |
|  | Beech / Spruce | 1.39 | 0.15–13.33 | 0.7726 | 0.7726 |
|  | Birch / Hornbeam | 43.68 | 2.27–841.14 | 0.0123 | 0.0370 |
|  | Birch / Spruce | 13.31 | 3.80–46.62 | 0.0001 | 0,0003 |
|  | Hornbeam/Spruce | 0.30 | 0.02–5.95 | 0.4330 | 0.5196 |
| bacterial *amoA* | Beech / Birch | 24.00 | 6.23–92.52 | <0.0001 | <0.0001 |
|  | Beech / Hornbeam | 660.29 | 93.43–4666.61 | <0.0001 | <0.0001 |
|  | Beech / Spruce | 1278.60 | 316.86–5159.38 | <0.0001 | <0.0001 |
|  | Birch / Hornbeam | 27.52 | 3.72–203.62 | 0.0012 | 0.0014 |
|  | Birch / Spruce | 53.28 | 12.39–229.17 | <0.0001 | <0.0001 |
|  | Hornbeam / Spruce | 1.94 | 0.25–14.78 | 0.5200 | 0.5200 |
| Archaeal *amoA* | Beech / Birch | 0.30 | 0.01–8.37 | 0.4820 | 0.5784 |
|  | Beech / Hornbeam | 14.64 | 0.21–1002.82 | 0.2134 | 0.4001 |
|  | Beech / Spruce | 6.46 | 0.24–173.68 | 0.2667 | 0.4001 |
|  | Birch / Hornbeam | 48.06 | 0.73–3167.57 | 0.0700 | 0.2099 |
|  | Birch / Spruce | 21.20 | 1.04–431.76 | 0.0470 | 0.2099 |
|  | Hornbeam / Spruce | 0.44 | 0.01–28.39 | 0.7002 | 0.7002 |
| *nxrB* | Beech / Birch | 3.41 | 0.29–40.82 | 0.3324 | 0.3988 |
|  | Beech / Hornbeam | 72.77 | 2.33–2269.80 | 0.0146 | 0.0292 |
|  | Beech / Spruce | 85.36 | 7.09–1027.68 | 0.0005 | 0.0014 |
|  | Birch / Hornbeam | 21.32 | 0.81–558.37 | 0.0663 | 0.0994 |
|  | Birch / Spruce | 25.02 | 6.28–99.67 | <0.0001 | <0.0001 |
|  | Hornbeam / Spruce | 1.17 | 0.05–30.77 | 0.9237 | 0.9237 |

Table S3. Generalized linear mixed models (Gamma, log link) evaluating tree species effects on N-cycling genes, nutrient content and N_2_O dynamics. Marginal R² represents variance explained by species; conditional R² includes location random effects.

| **Tree tissues** | **Analysis** | **Marginal R²** | **Conditional R²** | **Random effect variance** |
| --- | --- | --- | --- | --- |
| Shoot | Ammonia | 0.75 | 0.76 | 0.01 |
|  | Nitrite | 0.70 | 0.77 | 0.07 |
|  | Nitrate | 0.71 | 0.72 | 0.01 |
|  | N_2_O exchanges | 0.40 | 0.59 | 0.19 |
|  | *nirK* | 0.91 | 0.97 | 0.06 |
|  | *nirS* | 0.97 | NA* | NA |
|  | *norB* | 0.97 | NA* | NA |
|  | *nosZ* clade I | 0.92 | NA* | NA |
|  | *nosZ* clade II | 0.85 | 0.94 | 0.09 |
|  | bacterial *amoA* | 0.94 | NA* | NA |
|  | archaeal *amoA* | 0.72 | 0.95 | 0.23 |
|  | *nxrB* | 0.84 | 0.94 | 0.10 |
| Wood cores | Ammonia | 0.11 | NA* | NA |
|  | Nitrite | 0.10 | NA* | NA |
|  | Nitrate | 0.09 | NA* | NA |
|  | $\Delta$ N_2_O concentration | 0.29 | 0.79 | 0.50 |
|  | *nirK* | 0.39 | 0.91 | 0.52 |
|  | *nirS* | 0.42 | 0.92 | 0.50 |
|  | *norB* | 0.85 | NA* | NA |
|  | *nosZ* clade I | 0.36 | 0.90 | 0.54 |
|  | *nosZ* clade II | 0.34 | 0.79 | 0.46 |
|  | bacterial *amoA* | 0.88 | NA* | NA |
|  | archaeal *amoA* | 0.17 | 0.88 | 0.71 |
|  | *nxrB* | 0.52 | 0.87 | 0.35 |

* Conditional R² could not be estimated due to singular random-effect structure


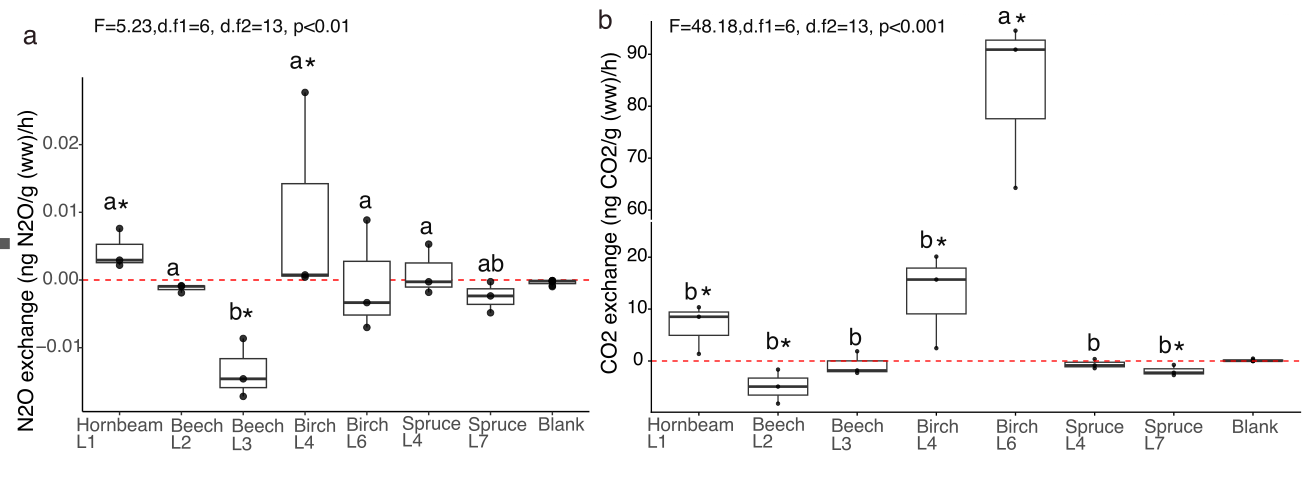
Figure S3: N_2_O concentration in stem wood (C_stem wood_ – C_ambient air_; mean–standard deviation). Significant differences are indicated by different letters (*P* < 0.05; dunn’s test) between different locations. L1= Lanžhot, Czech Republic; L2= Štítná nad Vláří, Czech Republic; L3= Bromarv, Finland; L4=Agali, Estonia; L5= Kiidjärve, Estonia; L6=Puijo, Finland; L7=Kenttärova-Pallas, Finland.


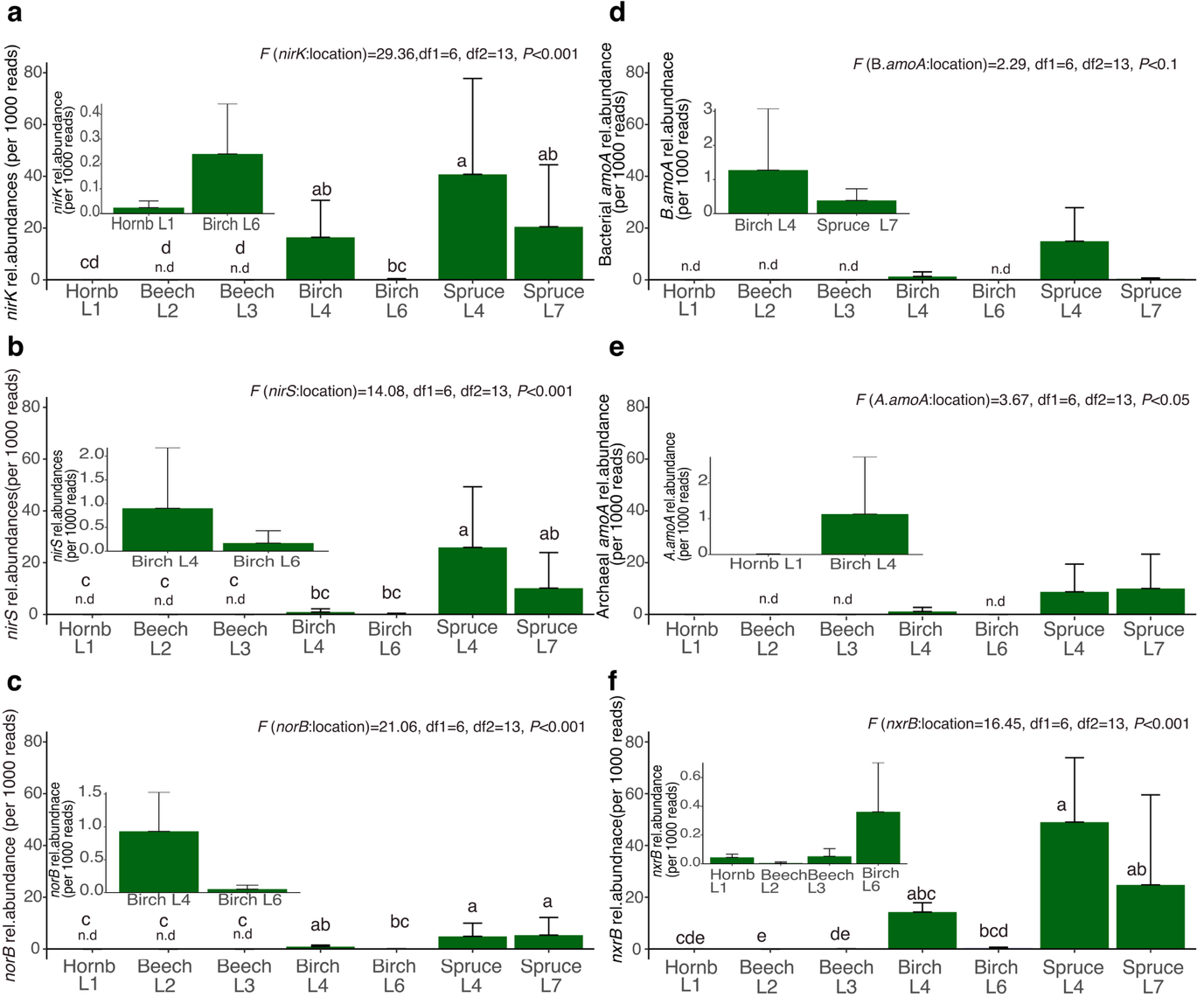


Figure S4: Relative abundances of denitrification and nitrification genes in shoot tissues from different locations. Relative abundances were calculated as total counts in relation to total reads (mean+standard deviation). (a) *nirK*, (b) *nirS*, (c) *norB*, (d) bacterial *amoA*, (e) archaeal *amoA*, (f) *nxrB*. Sites where N cycling genes were not detected are marked with n.d. L1= Lanžhot, Czech Republic; L2= Štítná nad Vláří, Czech Republic; L3= Bromarv, Finland; L4=Agali, Estonia; L6=Puijo, Finland; L7=Kenttärova-Pallas, Finland.


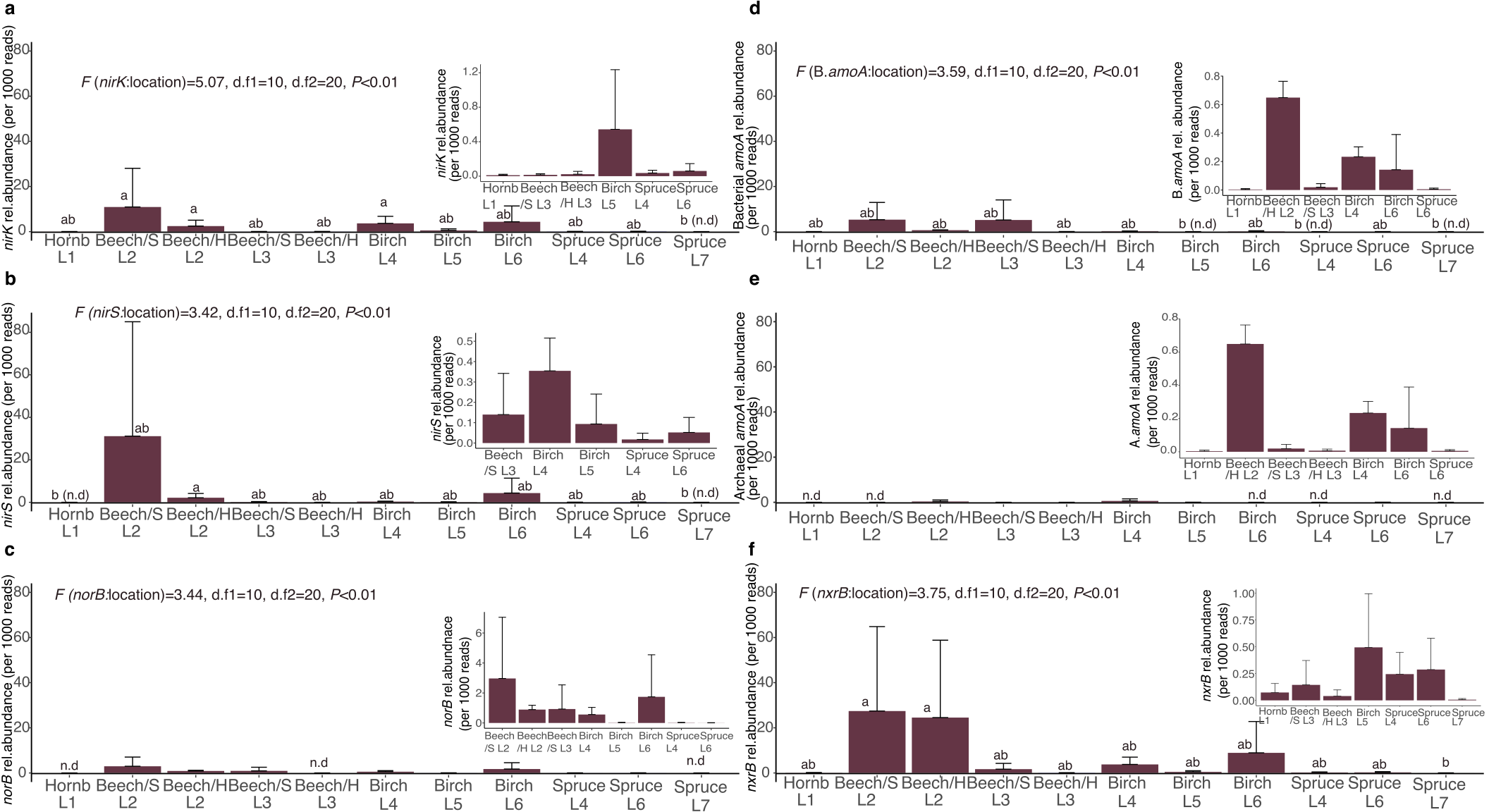


Figure S5: Relative abundances of denitrification and nitrification genes in wood cores from different locations. Relative abundances were calculated as total counts in relation to total reads (mean+standard deviation). (a) *nirK*, (b) *nirS*, (c) *norB*, (d) bacterial *amoA*, (e) archaeal *amoA*, (f) *nxrB*. Sites where N cycling genes were not detected are marked with n.d. Beech/S means beech sapwood and Beech/H means beech heartwood. L1= Lanžhot, Czech Republic; L2= Štítná nad Vláří, Czech Republic; L3= Bromarv, Finland; L4=Agali, Estonia; L5= Kiidjärve, Estonia; L6=Puijo, Finland; L7=Kenttärova-Pallas, Finland.


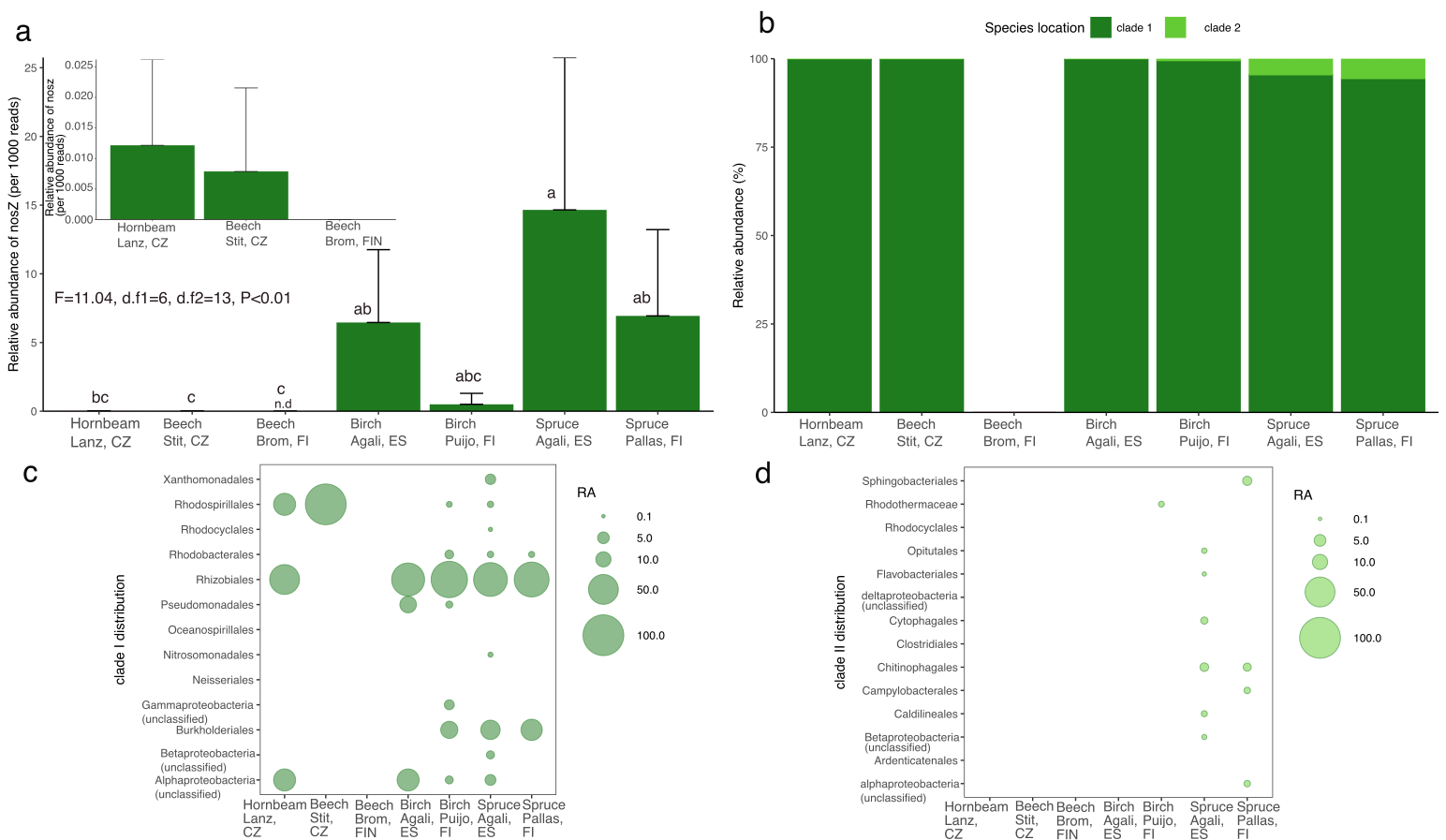


Figure S6: The *nosZ* genes in shoots. (a): Relative abundance of *nosZ* genes calculated as total counts in relation to total reads (mean+standard deviation). (b): Clade-specific relative abundance of *nosZ* normalized to total *nosZ* reads. (c) and (d): *nosZ* clade I and II distribution based on the classification at the order level. Sites where N cycling genes were not detected are marked with n.d. L1 = Lanžhot, Czech Republic; L2 = Štítná nad Vláří, Czech Republic; L3 = Bromarv, Finland; L4=Agali, Estonia; L6=Puijo, Finland; L7=Kenttärova-Pallas, Finland.


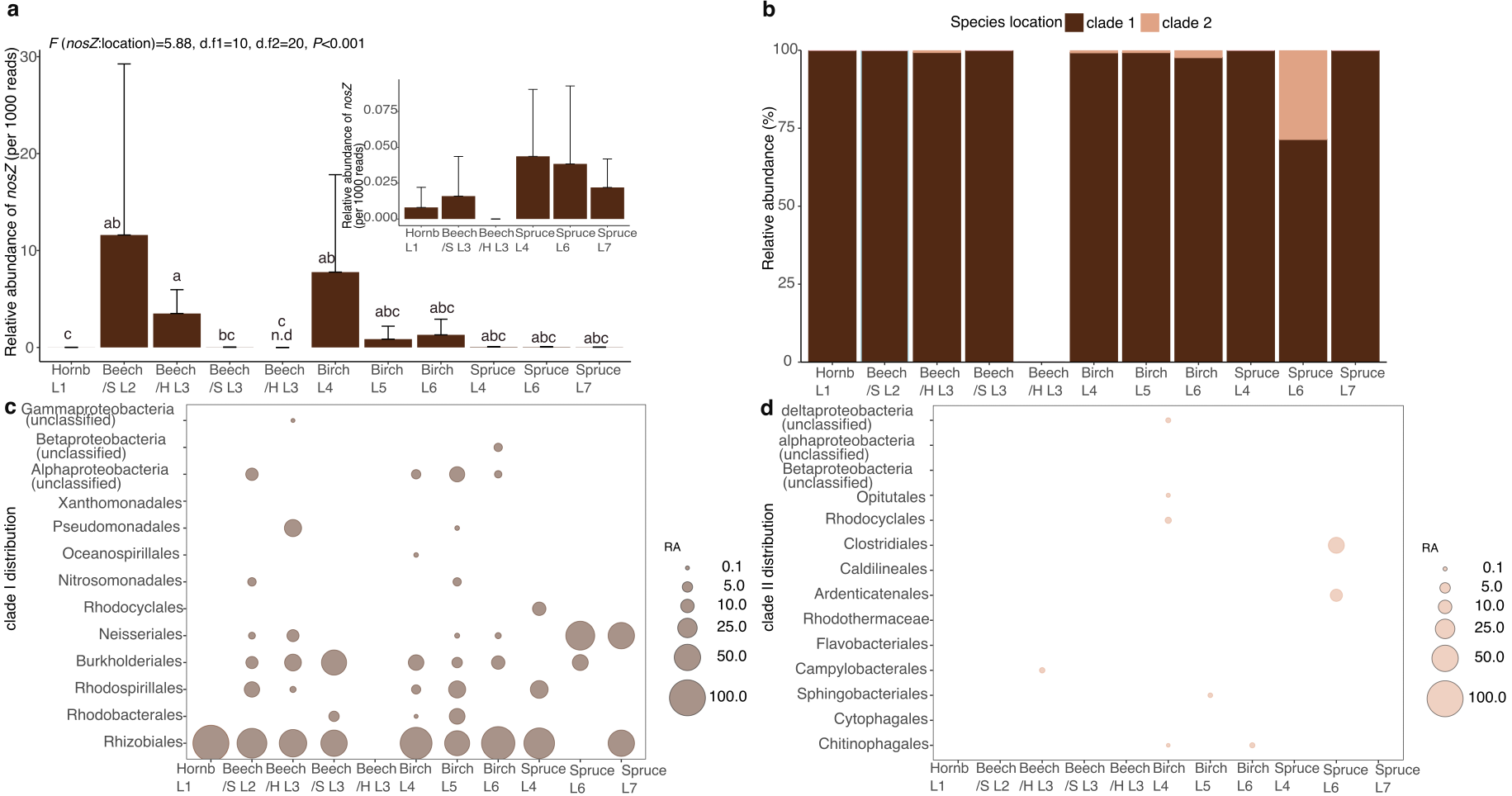


Figure S7: *nosZ* genes in wood core samples. (a): Relative abundance of *nosZ* calculated as total counts in relation to total reads (mean+standard deviation). (b): Clade-specific relative abundance of *nosZ* normalized to total *nosZ* reads. (c) and (d): *nosZ* clade I and II distribution based on the classification at the order level. Beech/S means beech sapwood and Beech/H means beech heartwood. Sites where N cycling genes were not detected are marked with n.d. L1= Lanžhot, Czech Republic; L2= Štítná nad Vláří, Czech Republic; L3= Bromarv, Finland; L4=Agali, Estonia; L5= Kiidjärve, Estonia; L6=Puijo, Finland; L7=Kenttärova-Pallas, Finland.


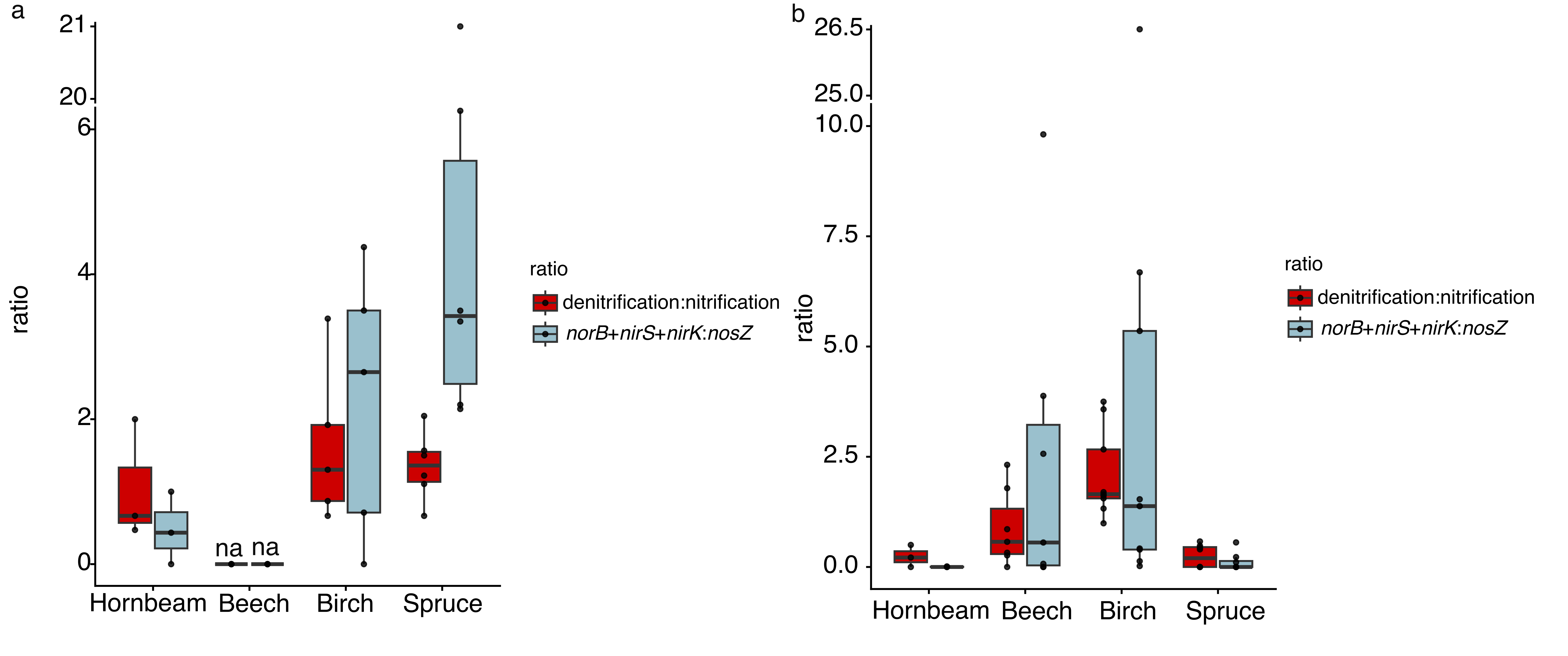


Figure S8. Ratio of N‑cycling genes in a) shoots and b) wood cores across tree species. “na” indicates values that could not be calculated because the denominator was zero.
